# Facilitators and barriers to the use of a personalised digital decision aid in total knee replacement consultations: insights from patients and orthopaedic surgeons – an interview study

**DOI:** 10.1186/s12913-025-13351-y

**Published:** 2025-10-21

**Authors:** Stefanie Deckert, Julia Slesaczeck, Diana Druschke, Franziska Beyer, Jochen Schmitt, Jörg Lützner, Toni Lange

**Affiliations:** 1 Center for Evidence-Based Healthcare, Faculty of Medicine, University Hospital Carl Gustav Carus, TUD Dresden University of Technology, Dresden, Germany; 2 Quality and Medical Risk Management, University Hospital Carl Gustav Carus, TUD Dresden University of Technology, Dresden, Germany; 3 Institute of Health and Nursing Science, Medical Faculty of Martin Luther University Halle-Wittenberg, University Medicine Halle, Halle (Saale), Germany; 4 University Center of Orthopaedics, Trauma and Plastic Surgery, University Hospital Carl Gustav Carus, TUD Dresden University of Technology, Dresden, Germany

**Keywords:** Total knee replacement, Indication, Practice guideline, Decision aid, Implementation

## Abstract

**Background:**

A substantial proportion of patients remain dissatisfied following total knee replacement (TKR), possibly due to a lack of standardised indication criteria and inadequate incorporation of patients’ preoperative expectations. Therefore, a practice guideline for TKR indication was developed to ensure the careful selection of patients suitable for this procedure. Based on this guideline, we developed a personalised digital decision aid (EKIT tool) to support implementing its recommendations and to improve shared decision-making (SDM) in routine care. This study explored patients’ and orthopaedic surgeons’ perceptions of the EKIT tool’s facilitators and barriers.

**Methods:**

This descriptive qualitative study was nested in a randomised controlled trial that assessed the effectiveness of the EKIT tool. We conducted semi-structured telephone interviews with patients considering TKR and orthopaedic surgeons, focusing on themes such as usability, comprehensibility, and perceived usefulness, which we used to identify facilitators and barriers of the EKIT tool. The interviews were transcribed verbatim and analysed using qualitative content analysis.

**Results:**

Seventeen participants were interviewed: nine patients and eight orthopaedic surgeons. Both groups highlighted the EKIT tool’s positive impact on SDM as a major facilitator. The orthopaedic surgeons emphasised that the EKIT tool enhanced the implementation of a structured, patient-centred and guideline-based consultation, facilitated clearer communication regarding patients’ expectations and included a thorough consideration of their possible achievement by TKR. The patients also emphasised the importance of addressing individual expectations during consultations. Those patients who had already made their decision before the consultation reported lower perceived usefulness of the EKIT tool but felt validated and more confident in their decisions. Main barriers reported by the orthopaedic surgeons were the time required and patient-related factors such as heterogeneous information needs and moderate health literacy. Some patients felt overwhelmed by the amount of health information provided.

**Conclusions:**

The EKIT tool has great potential for patients and orthopaedic surgeons to improve SDM in TKR consultations, appears to increase an informed decision for patients who are undecided, and increases confidence for patients who have already decided. All barriers can be addressed, leading to an optimised EKIT tool.

**Supplementary Information:**

The online version contains supplementary material available at 10.1186/s12913-025-13351-y.

## Background

Total knee replacement (TKR) is one of the most common orthopaedic surgical procedures and effectively improves pain, function, and quality of life for the majority of patients when non-surgical treatments have failed [1]. Advanced knee osteoarthritis (OA) is the main clinical indication for TKR [1]. Due to an ageing and increasingly obese population, the prevalence of knee OA continues to grow worldwide, which will tie up immense costs and capacities in the healthcare system [2]. Despite its benefits, a significant proportion of patients remain dissatisfied following a TKR [3]. In addition to complications and residual symptoms, other factors contributing to patient dissatisfaction after TKR are the lack of standardised indication criteria [4–6], inadequate consideration of patients’ preoperative expectations, and insufficient assessment of whether these expectations are achievable by TKR [7–9]. These issues impede orthopaedic surgeons from carefully selecting suitable patients with knee OA for TKR.

Therefore, standardised indication criteria and close patient involvement to guide shared decision-making (SDM) are warranted. SDM is a collaborative process between the patient, who provides insights into their expectations, values, and preferences, and the clinician, who outlines the benefits, risks, and uncertainties of various treatment options based on their experience and knowledge of the best available research evidence and recommendations [10, 11]. The benefits of SDM for patients with knee OA considering TKR have been demonstrated to include improved health outcomes, greater treatment satisfaction, improved decision quality, and a greater likelihood of not regretting their decision [12–15].

To address the aforementioned needs, the German initiative ‘*Evidenz- und konsensbasierte Indikation Knie-Totalendoprothetik*’ (EKIT; English: ‘evidence- and consensus-based indication criteria for total knee arthroplasty’) [5] developed a practice guideline entitled ‘Indication for Total Knee Replacement’ [16, 17]. This consensus-driven guideline incorporates both scientific evidence on the appropriateness of TKR criteria and patients’ preoperative expectations. The key outcome of this guideline was establishing minimum indication requirements to improve the selection of candidates for TKR [16, 17]. In order to support the implementation of these guideline-based indication criteria and promote SDM in routine care, the EKIT initiative developed a personalised digital decision aid: the EKIT tool. The Value-based TKR multicentre, stepped-wedge cluster randomised controlled trial (ClinicalTrials.gov: NCT04837053; registration date: 2021-04-07) evaluated the impact of a TKR consultation with and without the EKIT tool on decision quality (primary outcome) in routine care [18]. For this trial, 10 high-volume orthopaedic clinics (study centres) in Germany were cluster randomised. The intervention group received TKR consultation using the app-based EKIT tool, while the control group received TKR consultation according to the individual standard of clinical practice of each study centre (routine care).

This study aimed to explore both patients’ and orthopaedic surgeons’ perspectives on the EKIT tool’s facilitators and barriers for TKR consultation.

## Methods

### Study design

This was a nested descriptive qualitative study using semi-structured telephone interviews with patients and orthopaedic surgeons involved in the Value-based TKR trial. The detailed methods of the Value-based TKR trial have been previously published [19]. The qualitative study presented here was approved by the Ethics Committee of the Faculty of Medicine of the Technical University of Dresden, Germany (approval number EK-271062020) and was conducted in compliance with the Declaration of Helsinki. Reporting was guided by the Standards for Reporting Qualitative Research checklist [20] (Additional file1).

### Description of the EKIT tool

The EKIT tool was developed in 2021 according to international standards for patient decision aids [21, 22] in collaboration with clinical experts, healthcare researchers, and IT specialists. Its usability was tested in advance with appropriate patients and orthopaedic surgeons.

**Fig. 1:**
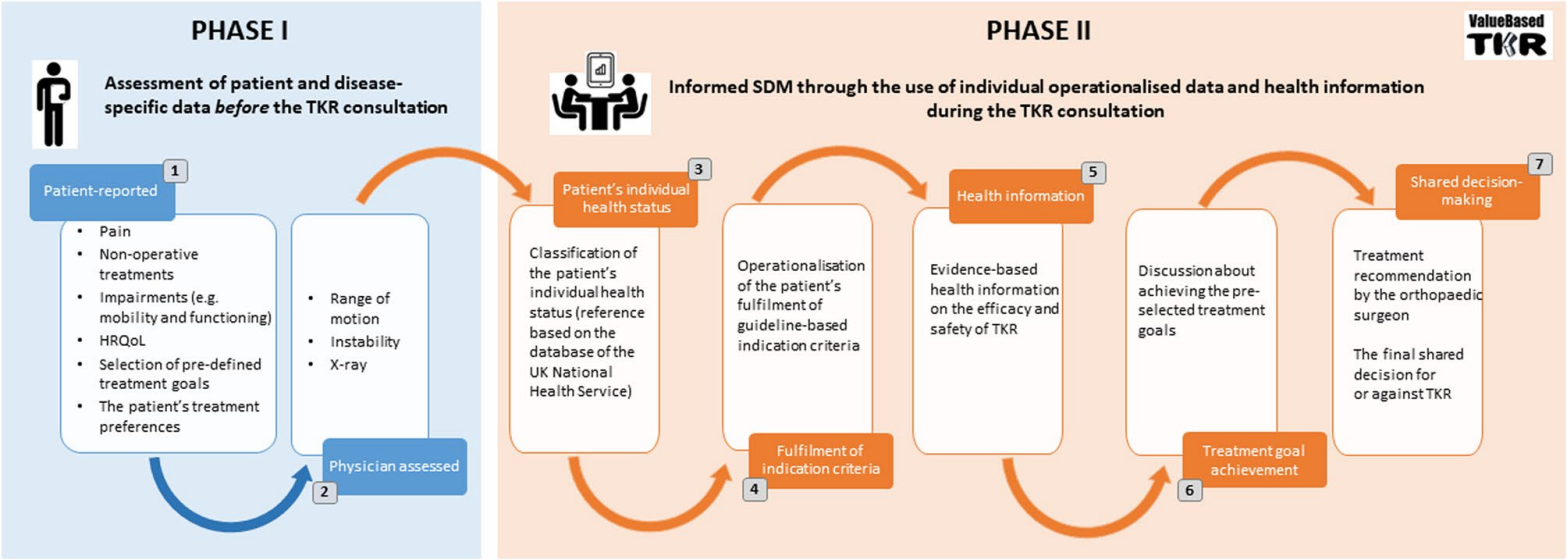
Structure and content of the EKIT tool

The EKIT tool is structured in two phases (Fig. 1). *Phase I* collects patient and disease-specific data immediately before the TKR consultation [16, 19]. In *Phase II*, which occurs during the consultation, the individual operationalised results of the data collected in *Phase I* are presented to the patient and orthopaedic surgeon, evidence-based health information on the efficacy and safety of TKR is explained [23], and the orthopaedic surgeon’s appraisal of how likely TKR is to achieve the patient’s treatment goals is discussed. Finally, the EKIT tool aims to enable SDM leading to a final patient-centred decision in favour of TKR or other operative or non-operative treatments (e.g. physical therapy). Figure 1 provides a detailed overview of the EKIT tool’s structure and content. Screenshots of its user interface are shown in Additional File 2.

In *Phase I*, the patients entered data using a tablet and orthopaedic surgeons using a computer. Patients had access to study staff support at all times. In *Phase II*, visualisations of the EKIT tool on the computer screen supported the TKR consultation, which was conducted in a consulting room.

### Participants

This study planned to recruit 20 interview partners from the 10 study centres participating in the Value-based TKR trial: one patient and one orthopaedic surgeon per study centre. The participants were recruited using a selective sampling strategy to obtain a heterogeneous cohort.

Our qualitative sampling plan for patient recruitment was based on literature and clinical expertise. It considered sex, the average age at which patients undergo their first TKR [24], and the percentage of patients who opted for or against TKR. In addition to the Value-based TKR trial’s inclusion criteria (diagnosed with knee OA, candidate for TKR, aged ≥ 18 years, cognitively unimpaired, able to speak German, and able to provide informed consent), we included only patients from the intervention group who had not previously undergone a TKR on the respective knee, were willing to participate, and were able to provide informed consent. In cooperation with study staff at each study centre, we identified and approached eligible patients following TKR consultations that used the EKIT tool to participate in this study. Eligible patients received the patient information leaflet of this study, and the study team contacted interested patients via telephone or e-mail. All patients who were contacted agreed to participate in the interview study and were included by the study team. Based on the number of patients included, the study team regularly provided a list of those still missing, in line with our sampling plan. This enabled study staff at each centre to approach consecutively. Since the interviews were conducted after using the EKIT tool, the patients had to recall its contents. In order to limit recall bias, patient interviews were conducted within two weeks of using the EKIT tool, and patients received screenshots of the EKIT tool as a reminder.

For orthopaedic surgeons, we considered age, sex, and professional position. Only those with sufficient experience using the EKIT tool were eligible to participate in this study (i.e. they needed to have used the EKIT tool with at least 20 patients and for at least four weeks). If more than one orthopaedic surgeon at a study centre met the inclusion criteria, we selected the participant according to our sampling plan.

All participants (patients and orthopaedic surgeons) provided written informed consent to participate in this study and were offered no incentives to participate.

### Data collection

**Fig. 2:**
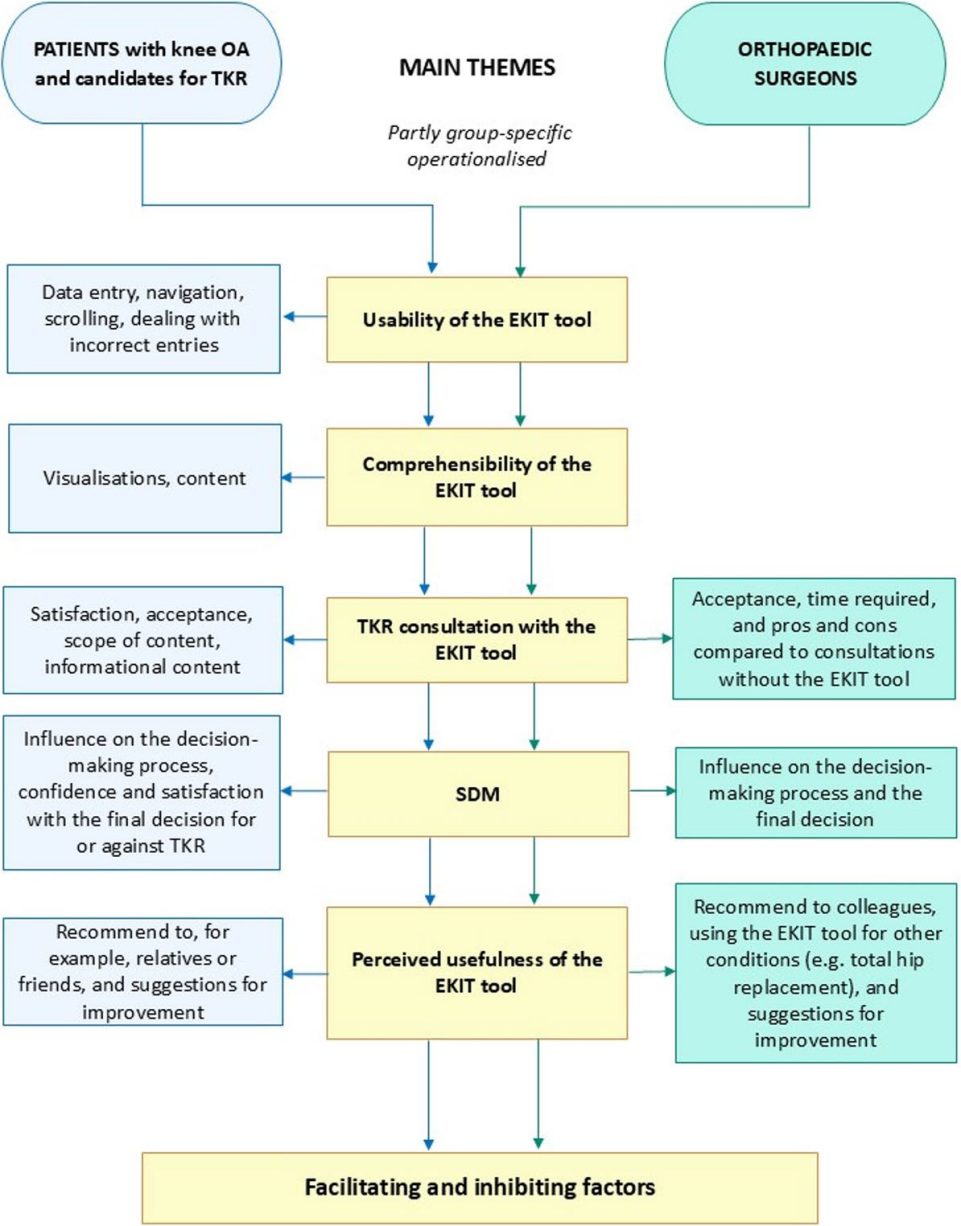
The main themes and sub-themes of the interview guides for the patients and orthopaedic surgeons

An experienced qualitative researcher designed the semi-structured interview guide for each interview group based on SDM concepts [25–29] and the Technology Acceptance Model [30]. Each guide consisted of five main themes: usability, comprehensibility, satisfaction with TKR consultation, SDM, and perceived usefulness. Experienced researchers in knee OA and TKR reviewed and modified both interview guides. We also conducted two preparatory interviews to familiarise ourselves with and review the guides: one with a patient and one with an orthopaedic surgeon with over 25 years of experience in TKR. The pilot testing resulted in minor modifications to the interview guides. As intended, the semi-structured guides provided orientation for the interviewer but still allowed for thematic digressions. The final structure and main themes of the guides are shown in Fig. 2. Therefore, exploring experiences with the EKIT tool, all themes were addressed in both interview groups, with some group-specific operationalisation. The interviews ended with standardised questions about the participant’s demographics. The patients were asked about their sex, age, and education, while orthopaedic surgeons were asked about their sex, age, and professional position. The final versions of the group-specific interview guides are provided in Additional files 3 (patients) and 4 (orthopaedic surgeons).

Given the stepped wedge design of the multicentre Value-based TKR trial, data collection for this study occurred from March 2022 to April 2023. A well-trained and experienced female qualitative researcher conducted the interviews. There was no prior relationship between the interviewer and the participants. The participants did not know the interviewer personally; they only knew she was a researcher. The patients were interviewed by telephone, at home or in another convenient location. The orthopaedic surgeons were also interviewed by telephone at their workplace, at home, or while travelling on business. The interviews were conducted in German and recorded using an audio recording device.

### Data processing

The audio recordings of the semi-structured interviews were anonymised and transcribed verbatim by a third-party staff member. The participants were not involved in data processing or analyses. We did not return the transcripts to the interviewees for comments. Before analysing the interviews, one researcher developed data codes (themes) based on the interview guide, and another reviewed these codes for accuracy and completeness. The themes were defined as follows: (i) usability, (ii) comprehensibility (content and visualisation), (iii) TKR consultation, (iv) SDM, (v) perceived usefulness of the EKIT tool, and (vi) participants’ characteristics and general data.

All but the last theme were subsequently used to operationalise the EKIT tool’s facilitators and barriers (Fig. 2).

### Data analysis

The participants**’** sociodemographic data were descriptively analysed using Microsoft Excel (version 2016). The transcribed interviews – including the pretest interviews – were analysed using the structured content analysis method developed by Mayring [31] with the MAXQDA software [32], to structure the collected data and create a coding system. The approach to developing codes was both deductive and inductive. First, both analysts read the transcribed interviews to become familiar with the content. Ambiguous passages were clarified by reviewing the audio recordings. Coding started with developing a deductive category system based on the Technology Acceptance Model and the a priori defined data codes derived by the interview guides (see Fig. 2), which were discussed and agreed upon within the study team. Based on these data codes, the transcripts were analysed and roughly coded. Subsequently, an inductive approach was applied by the development of additional categories and sub-codes. This was done by elaborating on emerging themes in the material and compiling sub-codes under the existing data codes. The primary analyst systematically applied the consolidated coding frame to all transcripts. The code systems developed for both interview groups are presented in Additional files 5 (patients) and 6 (orthopaedic surgeons). We defined coding rules and code specifications for each code and sub-code.

A second analyst again reviewed the assignment of individual text passages to the respective categories. Differences in coding were discussed and agreed upon between both analysts. When necessary, text segments were recorded accordingly. For data analysis, both manifest and latent content were considered. Finally, the main categories were used to synthesise the facilitating and inhibiting factors. If saturation were not achieved with our predefined sample size, recruitment would continue until no new analytical theme emerged.

The three female researchers involved (aged between 26 and 42) had prior experience with qualitative methods to varying degrees and had backgrounds in psychology (master’s degree), public health (doctorate) and nursing (master’s degree). None of them had a personal interest in the EKIT tool or experience of treating patients with knee OA. A native speaker translated the quotations into the English language for publication.

## Results

### Participants

Semi-structured interviews were conducted with 17 participants: nine patients (mean age: 66 ± 9.5; five females) and eight orthopaedic surgeons (mean age: 39 ± 8.1; three females). The study staff recruited patients from six of the 10 participating German study centres and orthopaedic surgeons from eight. There were no dropouts. Table 1 presents the characteristics of the participating patients and orthopaedic surgeons. The interviews lasted 22–54 min in both groups, with a mean duration of 32 min. The five themes related to our interview guide are described in turn below.

**Table 1:** Participants’ characteristics

	Patients (*n* = 9)	Orthopaedic surgeons* (*n* = 8)
Age (years), mean ± SD	66 ± 9.5	39 ± 8.1^#^
Sex (female), *n*	5	3
Education, *n*	High: 1 Middle: 7 Low: 1	High: 8
Professional experiences (years)		
mean ± SD	NA	16 ± 5.45
median (min; max)		14 (11; 27)
Time since knee complaints (years), mean ± SD	10.1 ± 5.38	NA
SDM after TKR consultation, *n*	For TKR: 7 Against TKR: 2	NA

* One participant was a physician assistant who applied the EKIT tool in the respective study centre;

^#^ Age was not reported by one orthopaedic surgeon

*Abbreviations*: *OA* osteoarthritis, *TKR* total knee replacement, *SDM* shared decision-making

### Themes

#### Usability

This theme relates to the data entry process during *Phase I* of the EKIT tool (Fig. 1). The patients and the orthopaedic surgeons both had positive experiences using the EKIT tool and were satisfied with it.

##### Patients

Of the nine patients, three entered all the data independently, two received partial assistance from the study staff, and four needed study staff to complete the data entry by reading each question aloud. Common reasons for needing assistance included a lack of knowledge and experience using a tablet, visual impairments, or finger stiffness. Patients who entered their data independently rated navigation, scrolling, ticking, and dealing with incorrect entries as easy to use. Since data entry procedures varied across study centres, the patients answered the questions in the waiting room or a separate room. Two of those who answered them in the waiting room reported that the crowded environment made it difficult to answer all questions in a focused manner.

##### Orthopaedic surgeons

Regarding the reasons for supporting patients with data entry, some orthopaedic surgeons highlighted time constraints as a key factor, particularly due to the Value-based TKR trial being conducted in routine care with tightly scheduled consultations. They also mentioned that using a computer for data entry (clinical examination results, see Fig. 1) was simple.

#### Comprehensibility

This theme relates to both phases of the EKIT tool (Fig. 1). Overall, most participants in both groups agreed that the EKIT tool’s visualisation (tables, figures, and diagrams) and content (texts and formulated questions) were comprehensible (Additional file 2).

##### Patients

Three of the nine patients stated that they found it difficult to understand the illustrations and diagrams because they were unfamiliar with such graphics (Additional file 2, slide 3), with one stating:

*‘It’s confusing for me at first. To be honest*,* I don’t know what it means. Initially*,* it overwhelms me; I either have to deal with it or have someone explain it to me. The terms frequency*,* minimum*,* and optimum* [in the diagram] *– I’m not familiar with them.’* (male patient, 61 years).

Some patients felt overwhelmed by the volume of information, particularly when choosing their treatment goals, and wished they had more time for this part of the EKIT tool. They were rarely or never explicitly asked about their treatment goals during medical consultations. Most patients had not thought about their expectations in this specific way beforehand.

##### Orthopaedic surgeons

The orthopaedic surgeons highlighted similar aspects to those of the patients regarding the comprehensibility of the EKIT tool. They highlighted that the illustrations and diagrams often caused comprehension problems for patients during consultations. Some perceived a lack of standardised guidance on how to explain the illustrated results of specific outcome measurement instruments as a personal barrier, leading to uncertainty in using this component of the EKIT tool.

#### TKR consultation

This theme relates to using the EKIT tool during the TKR consultation (*Phase II* of the EKIT tool). Based on the interview transcripts, we coded various aspects when evaluating feedback about the TKR consultation. The patients focused primarily on their overall satisfaction, whereas the orthopaedic surgeons focused on the EKIT tool’s impact on communication and the time required compared to their regular consultations without the EKIT tool.

##### Patients

Most patients expressed satisfaction with the TKR consultation and felt well-informed about their fulfilment of guideline-based indication criteria and the efficacy and safety of TKR. In this context, they valued the realistic interpretation of their knee-related complaints, which was enhanced by comparing their health status to a reference population with end-stage knee OA [33]. They also appreciated the EKIT tool’s encouragement to explicitly consider their expectations for TKR and assess how likely this can be achieved through TKR. One stated:

*‘From the patient’s perspective*,* it might not be a bad idea to ask what they actually expect from the operation. What are their expectations for the procedure itself and for the recovery process? How do they envision things going after the operation?’* (male patient, 53 years).

However, two patients were unsatisfied with some parts of the consultation but could not articulate their specific concerns. One stated, *‘You waited a long time for such an appointment*,* and I didn’t think the result was great.’* (female patient, 54 years).

The focus on providing health information decreased the satisfaction of a few patients. One criticised the excessive emphasis on surgery rather than conservative therapy, perceiving the information about treatment options as being unbalanced. Two also felt that the information provided about surgery was overly detailed; they preferred less extensive education on TKR, as they were already candidates for this kind of surgery. These examples illustrate that some patients have different information needs, both in terms of volume and content.

##### Orthopaedic surgeons

Compared to TKR consultations conducted without the EKIT tool, most orthopaedic surgeons reported longer TKR consultations with the EKIT tool due to the need for detailed discussions about treatment goals and more in-depth explanations. However, a few did not notice a significant difference. Nonetheless, one felt the time required for the consultation might even shift to other stages of patient healthcare, explaining in more detail as follows:

*‘While the consultation with the EKIT tool was more time-consuming*,* it could potentially reduce the time needed for postoperative discussions*,* such as during ward rounds or follow-up visits*,* because patients would be informed better and more realistically about their treatment outcomes.*’(female orthopaedic surgeon, 39 years).

Regarding the impact of the EKIT tool on communication, five orthopaedic surgeons noted an improvement compared to consultations without the EKIT tool, while two reported no change and one reported a deterioration. Moreover, five orthopaedic surgeons particularly valued the structured and standardised counselling provided by the EKIT tool without making them feel externally constrained. One stated:

*‘I believe that all in all*,* this* [EKIT tool] *can lead to – I would say a more standardised discussion – the patient is made aware of the risks and benefits. I think that’s a good thing.’* (male orthopaedic surgeon, 36 years).

Most orthopaedic surgeons felt that the health information regarding TKR was explained and demonstrated in a patient-friendly manner, particularly benefiting those with lower health literacy, and facilitated communication. However, TKR consultations with the EKIT tool presented some challenges, as some orthopaedic surgeons struggled to address the varying levels of health literacy among patients and their different information needs.

#### SDM

This theme explores how the EKIT tool influenced the SDM, particularly the patients’ contributions and their confidence and satisfaction with the final treatment decision.

##### Patients

Seven of the nine patients felt confident and satisfied with their final decision for or against TKR. One stated, *‘I feel somehow empowered now when I say – OK*,* maybe I can wait a little bit longer.’ (*female patient, 69 years). There were no cases where the treatment preferred by the patient did not match the treatment recommended by the orthopaedic surgeon.

When asking the patients about their contributions to their final decision, only active and collaborative roles could be identified, as the following quotes illustrate: ‘*I definitely had a role because I agreed to the decision.’* (male patient, 61 years) and ‘*I* *would say I played a one hundred per cent role.’* (male patient, 80 years). However, two patients expressed uncertainty due to previous surgeries, fear of complications, and a general lack of confidence in the success of the procedure.

##### Orthopaedic surgeons

The orthopaedic surgeons were positive about patient involvement in SDM. However, they emphasised different aspects in this context. Firstly, they regarded the EKIT tool as an additional validation of their treatment recommendations, reassuring that the guideline-based indication criteria for TKR were being met.

‘*It’s also reassuring. I review the X-ray*,* assess the patient’s limited mobility*,* and I consider their level of suffering. The data recorded and visualised in the EKIT tool provides additional confirmation that the indication is well-supported.’* (female orthopaedic surgeon, 45 years).

In this context, one orthopaedic surgeon expressed that the EKIT tool *‘helped to address important issues that might otherwise have been overlooked’* (male orthopaedic surgeon, 39 years).

Secondly, another orthopaedic surgeon assumed the EKIT tool could reduce the number of dissatisfied patients after TKR, stating in this context:

*‘And the next major advantage is*,* of course*,* the goals*,* the expectations patients have set for themselves*,* because sometimes they are really unrealistic. The EKIT tool provides a resource with information and documentation about what they really can expect or whether and how they can achieve their own goals.’* (female orthopaedic surgeon, 39 years).

Thirdly, one orthopaedic surgeon praised the scientific soundness of the EKIT tool within SDM.

#### Perceived usefulness

This theme assessed perceived usefulness, a person’s subjective perception of the benefits or positive effects of using the EKIT tool.

##### Patients

Most patients highly rated the EKIT tool’s perceived usefulness, particularly when mentioning that it helped with their decision-making. One stated, *‘I would have been glad if I had had the same experience with my opposite knee* [a few years ago]*’* (female patient, 69 years).

When patients had already decided before the consultation, the perceived usefulness of the EKIT tool on SDM was initially reduced. Further analysis revealed that six of the nine patients had already decided in favour of TKR before the consultation. One stated, *‘I went into the consultation room and said*,* I’m going to have the surgery; I’m not waiting any longer’* (female patient, 75 years). Another stated, *‘Yes*,* I also spend a considerable amount of time thinking about the operation in advance and gathering information. So*,* it wasn’t a spontaneous decision on my part.’* (female patient, 78 years).

Since these pre-consultation decisions aligned with the orthopaedic surgeons’ treatment recommendation after TKR consultation, all patients felt finally validated and more confident in their decision. Finally, all patients stated that they would recommend the EKIT tool as a decision aid for TKR to their relatives and friends and could envision using it for other treatment decisions in the future.

##### Orthopaedic surgeons

Almost all orthopaedic surgeons rated the EKIT tool as highly useful. Consequently, they would recommend it to colleagues and consider adapting it for other elective surgical procedures, such as total hip replacement. One even suggested that the EKIT tool should be ‘*a mandatory decision aid for all prosthetic specialists’* (male orthopaedic surgeon, 39 years). This positive feedback was nearly unanimous among all orthopaedic surgeons, although some highlighted certain limitations. Almost all orthopaedic surgeons felt that the usefulness of the EKIT tool was slightly limited, as a substantial proportion of patients had already made their decision before the consultation. Therefore, some suggested that the EKIT tool be utilised before referral for TKR, as this would likely be the most effective point in the decision-making process for patients. One stated:

*‘And things that frequently happen are that many patients are sitting there*,* and the decision about the total knee replacement has already been made. So it’s uncommon during the consultation that specific questions are asked again or that patients express a strong desire for additional information.’* (male orthopaedic surgeon, age not reported).

One improvement commonly suggested by the orthopaedic surgeons related to time; they recommended shortening the EKIT tool as much as possible to increase its perceived usefulness. Some also stated that incorporating risk factors and comorbidities into the EKIT tool would improve its usefulness. Moreover, its usefulness was somewhat limited by certain patient characteristics, such as age, language barriers, and cognitive impairment.

### Facilitating and inhibiting factors

The interviews revealed several facilitators and barriers related to the EKIT tool, which are listed in Table 2.

**Table 2:** EKIT tool’s facilitating and inhibiting factors based on patients’ and orthopaedic surgeons’ perspectives

	Patients	Orthopaedic surgeons
Facilitating factors	Support in defining and reflecting on their own treatment goals	Standardisation and structuring of the TKR consultation
	Orthopaedic surgeons are well-informed about their treatment preferences and expectations	Patient-friendly explanation of the benefits and risks of TKR, particularly for patients with low health literacy
	Improving understanding of their knee impairments through comparison with data from other patients with end-stage knee OA	Improved communication about patients’ expectations and how likely these are achievable after TKR
	Increase confidence in their final decision, also for those who had already decided in favour of TKR before the consultation	Further validation and assurance of the fulfilment of guideline-based indication criteria and considering patients’ treatment goals
		More active involvement of patients
Inhibiting factors	Technical challenges and individual barriers when using the tablet	Individual patient impairments, including visual impairments, finger stiffness, and cognitive or language barriers
	Feelings of being overwhelmed in selecting individual treatment goals	Longer TKR consultations
	Variability in needs for health information, including scope and volume, and insufficient explanations from orthopaedic surgeons about health information	Variability in patients’ information needs and health literacy
		Patients had already decided for or against TKR before the consultation

*Abbreviations*: *OA* osteoarthritis, *TKR* total knee replacement

#### Facilitating factors

From the perspective of both the patients and orthopaedic surgeons, the positive influence of the EKIT tool on SDM was the key facilitating factor. The patients indicated that the EKIT tool might positively influence SDM by encouraging them to define and critically reflect on their preoperative expectations, better understand and categorise their knee-related complaints, and become well-informed about the benefits and risks of TKR. From their perspective, an additional advantage of SDM is that orthopaedic surgeons gain insight into their expectations and treatment preferences. Consequently, using the EKIT tool empowered patients to carefully evaluate whether to proceed with TKR, leading to greater confidence in their final decision, regardless of whether they chose to undergo TKR.

The orthopaedic surgeons identified three key benefits of the EKIT tool: supporting and structuring the consultation, enhancing communication with patients, and its positive impact on SDM. In particular, the EKIT tool facilitated communication with patients, especially for those with low health literacy, and encouraged more effective discussions about patients’ expectations, leading to a more realistic understanding of anticipated treatment outcomes. In this context, they found the EKIT tool effectively revealed and clarified patient conflicts and appreciated the patient-friendly explanation of the benefits and risks of TKR. Regarding decision-making, the orthopaedic surgeons viewed the EKIT tool as an additional validation of their treatment recommendations, assuring that the guideline-based indication criteria for TKR were met. Based on the orthopaedic surgeons’ experiences with the EKIT tool, the patients were more actively involved in SDM.

#### Inhibiting factors

The orthopaedic surgeons considered the greater time commitment of TKR consultations with than without the EKIT tool to be the key barrier to using the EKIT tool. From their perspective, the varying levels of health literacy among patients and their different information needs (i.e. its scope and volume) were identified as two main inhibiting factors. These barriers to the EKIT tool largely mirrored those expressed by the patients, as some felt overwhelmed by the amount of health information provided, while others desired more information about various treatment options. The patients also expressed that some of the information presented through illustrations was not understood because they were unfamiliar with such forms of presentation, and the explanations given by the orthopaedic surgeons were insufficient for their understanding.

During data collection in *Phase I* of the EKIT tool, the patients reported several inhibiting factors to its usability, including the setting (e.g. a crowded waiting room), technical experiences, individual factors such as age, visual impairment, finger stiffness, and feelings of being overwhelmed when selecting individual treatment goals. These findings aligned with the orthopaedic surgeons, who identified similar barriers such as organisational issues (procedures specific to each clinic) and patient-related factors such as cognitive impairment, age, technical experience, and the need for support with data entry from study staff.

## Discussion

The EKIT tool is a personalised digital decision aid designed for use by patients and orthopaedic surgeons during TKR consultations. It aims to support the careful selection of suitable candidates for TKR. In terms of scope, target group, and data evaluation, there are no comparable decision aids available [13, 14, 19, 34–39]. The Value-based TKR trial already evaluated its effectiveness, showing that 86% of the patients whose consultations were conducted with the EKIT tool had good decision quality, compared to 67% of the patients whose consultations were conducted without the EKIT tool (relative risk: 1.24; 95% confidence interval: 1.15–1.33) [18].

Currently, no qualitative studies in the field of joint replacement surgery have explored both patients’ and orthopaedic surgeons’ perspectives on a specific decision aid [37, 40]. Patients’ views [41] and orthopaedic surgeons’ opinions [42] on a hypothetical decision aid have each been explored qualitatively only once. To further evaluate the EKIT tool, we conducted this qualitative study and interviewed both patients and orthopaedic surgeons, gaining insights into the facilitating and inhibiting factors of the EKIT tool.

Both patients and orthopaedic surgeons identified the EKIT tool’s positive impact on SDM as a key advantage. The orthopaedic surgeons noted that the EKIT tool supports a structured, patient-centred, and guideline-based consultation with explicit consideration and improved communication of patient expectations and an assessment of the likelihood of their achievement. This perspective aligns with the patients’ views, who emphasised the importance of discussing specific individual preoperative treatment goals on TKR. They also concluded that they were better informed about their health status and the risks and benefits of TKR and were more satisfied and confident with their final decision. These results are consistent with the systematic review by Stacey et al. [35], who demonstrated that decision aids increased knowledge, reduced decision-making conflicts and indecision, and improved communication. In an Australian interview study, orthopaedic surgeons believed decision aids could improve communication and patient informed consent [42], which could be shown in this qualitative study.

The barriers identified through our qualitative content analysis will allow the EKIT tool to be optimised, which will be discussed below.

### Optimal healthcare setting and scope of the EKIT tool

In Germany, recommendations concerning TKR are usually made by a general practitioner or orthopaedic specialist in an outpatient setting, with final decisions made during consultations with orthopaedic surgeons, often in inpatient settings. Therefore, the EKIT tool is used in the inpatient setting as a digital decision aid for SDM, clarifying the fulfilment of the indication criteria according to the German practice guideline [16] to support the final decision for or against TKR. However, some of our interviewed patients had already decided on TKR before their consultation, leading them to perceive the EKIT tool as less useful in influencing their decision-making. Nevertheless, they felt validated and more confident in their decision after the TKR consultation using the EKIT tool. Some orthopaedic surgeons considered these pre-consultation decisions to be a barrier to effective SDM, and some preferred implementing the EKIT tool at an earlier stage in the healthcare process (i.e. in the outpatient setting). Studies by Marshall et al. [14] and Barlow et al. [43] supported the orthopaedic surgeons’ preference, showing that patients who received information earlier in the care process found it more beneficial and influential in their decision-making.

As an additional inhibiting factor, some patients expressed concerns about receiving unbalanced information regarding non-surgical treatments and addressing their information needs. This perception may arise from the EKIT tool’s focus on clarifying indications for TKR in patients with advanced knee OA who are candidates for TKR. Consistent with the German practice guideline [16], TKR might not be indicated if non-surgical treatments, such as analgesics or physical therapy, have not been adequately addressed. In these cases, patients should receive appropriate non-surgical recommendations. However, this is only marginally addressed in the current version of the EKIT tool. To address patients’ needs for comprehensive information about non-surgical options, these should ideally be discussed in the outpatient setting, with referrals for TKR consultations only made after these non-surgical options have been thoroughly addressed. Orthopaedic surgeons in Australia also identified barriers, such as the lack of non-operative alternatives for managing end-stage knee OA, raising questions about the general utility of a decision aid for TKR [42].

In conclusion, the optimal healthcare setting for implementing the EKIT tool and its scope requires further discussion among various stakeholders, considering the structures of the German healthcare system.

### Using the EKIT tool before and during the TKR consultation

We identified patient-related factors as barriers, including different information needs, varying levels of health literacy, being overwhelmed when choosing preoperative treatment goals, and difficulty using a tablet. One way to address these barriers is to allow patients to complete the questionnaire (*Phase I* of the EKIT tool) and review the health information (*Phase II*) before their consultation at home using a PC or tablet. This approach could help patients reflect on their preoperative treatment goals, digest health information in a comfortable environment, and enable them to better prepare questions and clarify doubts for the TKR consultation, depending on their information needs. The early provision of certain components of the EKIT tool would also have the advantage of allowing relatives or friends to assist with data entry in a distraction-free environment with sufficient time for the patient and avoiding the inhibiting factors mentioned by the patients, such as finger stiffness and visual impairments. However, this approach could also pose challenges for patients with support needs. Therefore, how health information is made available and how health data is entered into the EKIT tool should allow for flexible options according to patients’ needs and skills. A subgroup analysis by Stacey et al. [44] found no difference in knowledge scores between patients who received health information before or during the consultation. However, receiving certain components of the EKIT tool beforehand may help patients feel more prepared and able to participate in the SDM process [45].

Irrespective of whether some components of the EKIT tool are used at home before the consultation or the entire EKIT tool is applied during the TKR consultation, it is essential to critically examine how the health information (e.g. provided by illustrations; see Additional File 2 [slides 1 and 2] for examples) can be made more understandable to laypeople. For example, providing orthopaedic surgeons with explanatory text or videos and standardised instructions for explaining the illustrations and diagrams could avoid any perception of limiting communication due to low health literacy.

Further revision of the EKIT tool should consider the possibility of providing components of the EKIT tool before the TKR consultation and the extent to which health information and its presentation can be adapted to different patient groups with different information needs and health literacy levels.

### Impact of the EKIT tool on the duration of TKR consultations

The orthopaedic surgeons also highlighted the time required for consultations with the EKIT tool as a barrier. The National Institute for Health and Care Excellence guideline states that appointments or consultations may take longer when using SDM [29]. A systematic review [35] found that using a decision aid extended consultation time by an average of 1.5 min compared to routine care. Therefore, it can be presumed that the perceived consultation time reported by our interviewed orthopaedic surgeons was shorter than they experienced. However, we cannot verify this assumption because the time required for a TKR consultation with and without the EKIT tool was not measured in the Value-based TKR study. Nevertheless, it is reasonable to assume that the first consultations with the EKIT tool took longer and that the time required for the consultation decreased as the number of consultations increased as the orthopaedic surgeons became more familiar with using it. Studies investigating the potential time-saving effects of SDM in the orthopaedic setting, such as a reduction in telephone calls, unnecessary clinic visits, discussions with dissatisfied patients due to unmet expectations, and cancelled operations, as anticipated by one interviewed orthopaedic surgeon, are still lacking [46], but would enrich the discussion.

The potential for time optimisation of the EKIT tool should be considered during its revision. Regardless, finding the optimal balance between patient-centred, high-quality care and the efficient use of time for patient care remains challenging for any healthcare system.

### Content-related adaptations of the EKIT tool

Patients who participated in focus group discussions in the United Kingdom expressed a desire to hear about other patients’ experiences before and after surgery [41]. We addressed this concern for preoperative experiences in the EKIT tool by comparing patients’ individual knee complaints with those of a reference population with advanced knee OA [33]. While postoperative experiences were not suggested as a potential facilitator of the EKIT tool in our study, incorporating this aspect using results from follow-up studies [8, 9] could provide valuable improvements to the EKIT tool and should be considered in any revision. Other issues raised by orthopaedic surgeons in our interviews, such as the assessment of risk factors and comorbidities, should also be considered.

### Adaptability of the EKIT tool to other elective surgical procedures

Almost all interviewed orthopaedic surgeons would recommend the EKIT tool to colleagues, to the point of considering it essential for all (elective) prosthetic specialists. Patients may also be interested in using such a tool for other types of consultations. However, interviews with orthopaedic surgeons in the United Kingdom revealed an attitude that suggested they believe they were already conducting these consultations effectively through their clinical reasoning – prioritising their clinical acumen – leading them to feel that a tool may not be necessary [42]. Wilson and Probe [46] found similar results, citing orthopaedic surgeons’ overconfidence and inadequate knowledge about SDM. Our study cannot verify these statements. However, because of its exploratory nature, we cannot rule out the possibility that other orthopaedic surgeons would not accept the EKIT tool in routine care. Willingness to use a decision aid in clinical practice must be investigated on a larger scale in the field of joint replacement surgery, such as via a market analysis.

### Limitations

A limitation of our study is that we did not reach our intended sample size. This shortfall was due to ongoing staff turnover and insufficient practical experience with the EKIT tool (i.e. fewer than 20 applications), which restricted the pool of eligible orthopaedic surgeons. The limited number of recruited patients in some centres also made finding patient participants for our study challenging. To address this, we interviewed two orthopaedic surgeons and four patients from one study centre to reach a sufficiently large sample, knowing that this approach may introduce selection bias. However, since our focus was on experiences with the EKIT tool rather than healthcare structures at individual centres, we do not anticipate this affected our results. This assumption is supported by the fact that we reached data saturation with the 17 transcripts we analysed. Another limitation of our study is that information on how many patients were approached by study staff at each study centre was not documented as part of the recruitment process. This means that a selection bias cannot be ruled out. Moreover, according to our predefined sampling criteria, older and female patients may have been underrepresented. Due to the retrospective design, the perspectives of both patients and orthopaedic surgeons were likely influenced by recall bias, despite our efforts to minimise the time between the consultation and the interview. It is possible, that more in-depth interviews, meaning with more open-ended questions, could have revealed further aspects. Finally, the transcripts were not returned to participants, as the interviews were clearly structured, and participants’ responses regarding the assessment of the EKIT tool were factual and clearly articulated. No sensitive experiences were shared that might have led to divergent interpretations.

## Conclusions

The EKIT tool has great potential to improve SDM between patients and orthopaedic surgeons in TKR consultations, increase informed decision-making by empowering those who are undecided, and increase confidence for those who have already decided. All explored barriers can be addressed, leading to an optimised EKIT tool. One of the most significant changes could be assessing whether some components of the EKIT tool can be used before the consultation (e.g. at home). This approach could minimise disruptions to hospital routines and give patients adequate time to engage with the EKIT tool. Before implementation, further discussion among stakeholders is essential to determine the optimal setting (inpatient and outpatient) and scope for integrating the EKIT tool into the German healthcare system. This discussion is especially significant since the EKIT tool has also been recommended for other joint replacement surgeries, and most orthopaedic surgeons favoured its widespread use and adoption.

## Supplementary Information

Additional file 1: Reporting checklist.

Additional file 2: Screenshots of user interfaces of the EKIT tool.

Additional file 3: Interview guide patients with knee osteoarthritis and candidates for TKR.

Additional file 4: Interview guide orthopaedic surgeons.

Additional file 5: Data coding system patients with knee osteoarthritis and candidates for TKR.

Additional file 6: Data coding system orthopaedic surgeons.

## Data Availability

The dataset generated and analysed during the current study are not publicly available because of data privacy concerns regarding the privacy and confidence of participants. The datasets, however, are available from the corresponding author upon reasonable request.

## Abbreviations

OA: Osteoarthritis
TKR: Total knee replacement
SDM: Shared decision-making
