# Supplementary material for: Facilitators and barriers to the use of a personalised digital decision aid in total knee replacement consultations: insights from patients and orthopaedic surgeons – an interview study
Source: BMC Health Serv Res. 2025 Oct 21;25:1387. doi: 10.1186/s12913-025-13351-y (PMC12541942; doi:10.1186/s12913-025-13351-y)

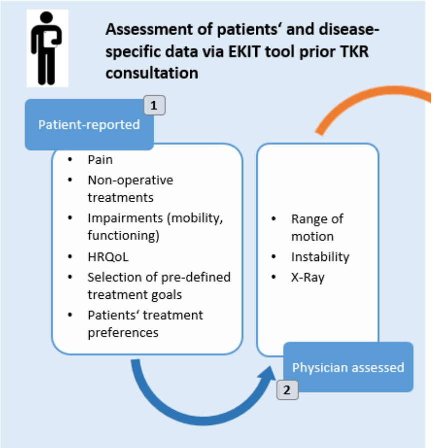
Assessment of patients' and disease-specific data via EKIT tool prior TKR consultation
1
Patient-reported
Pain
Non-operative treatments
Impairments (mobility, functioning)
HRQoL
Selection of pre-defined treatment goals
Patients' treatment preferences
Range of motion
Instability
X-Ray
Physician assessed
2

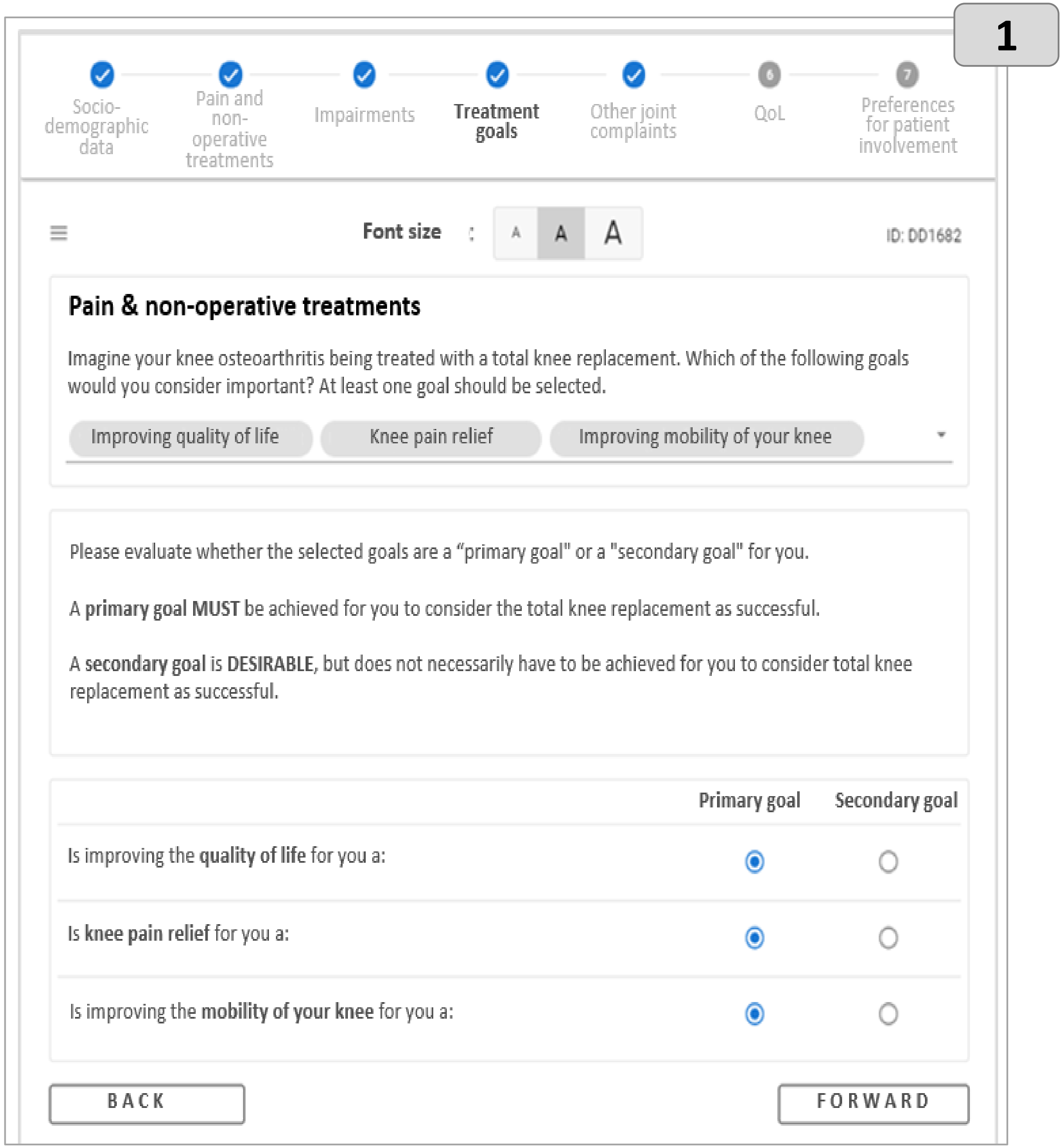
1
Socio-demographic data
Pain and non-operative treatments
Impairments
Treatment goals
Other joint complaints
6
QoL
7
Preferences for patient involvement
Font size :
A
A
A
ID: DD1682
Pain & non-operative treatments
Imagine your knee osteoarthritis being treated with a total knee replacement. Which of the following goals would you consider important? At least one goal should be selected.
Improving quality of life
Knee pain relief
Improving mobility of your knee
Please evaluate whether the selected goals are a "primary goal" or a "secondary goal" for you.
A primary goal MUST be achieved for you to consider the total knee replacement as successful.
A secondary goal is DESIRABLE, but does not necessarily have to be achieved for you to consider total knee replacement as successful.
Primary goal
Secondary goal
Is improving the quality of life for you a:
Is knee pain relief for you a:
Is improving the mobility of your knee for you a:
BACK
FORWARD

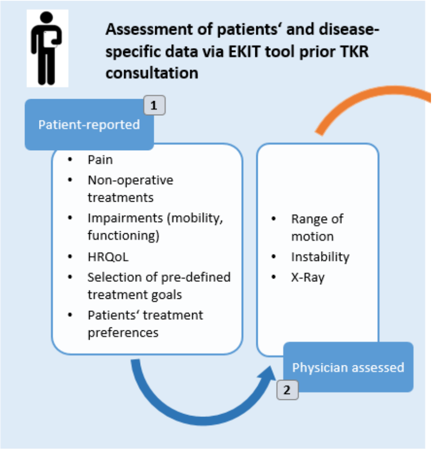

Assessment of patients' and disease-specific data via EKIT tool prior TKR consultation
Patient-reported
1
Pain
Non-operative treatments
Impairments (mobility, functioning)
HRQoL
Selection of pre-defined treatment goals
Patients' treatment preferences
Range of motion
Instability
X-Ray
Physician assessed
2

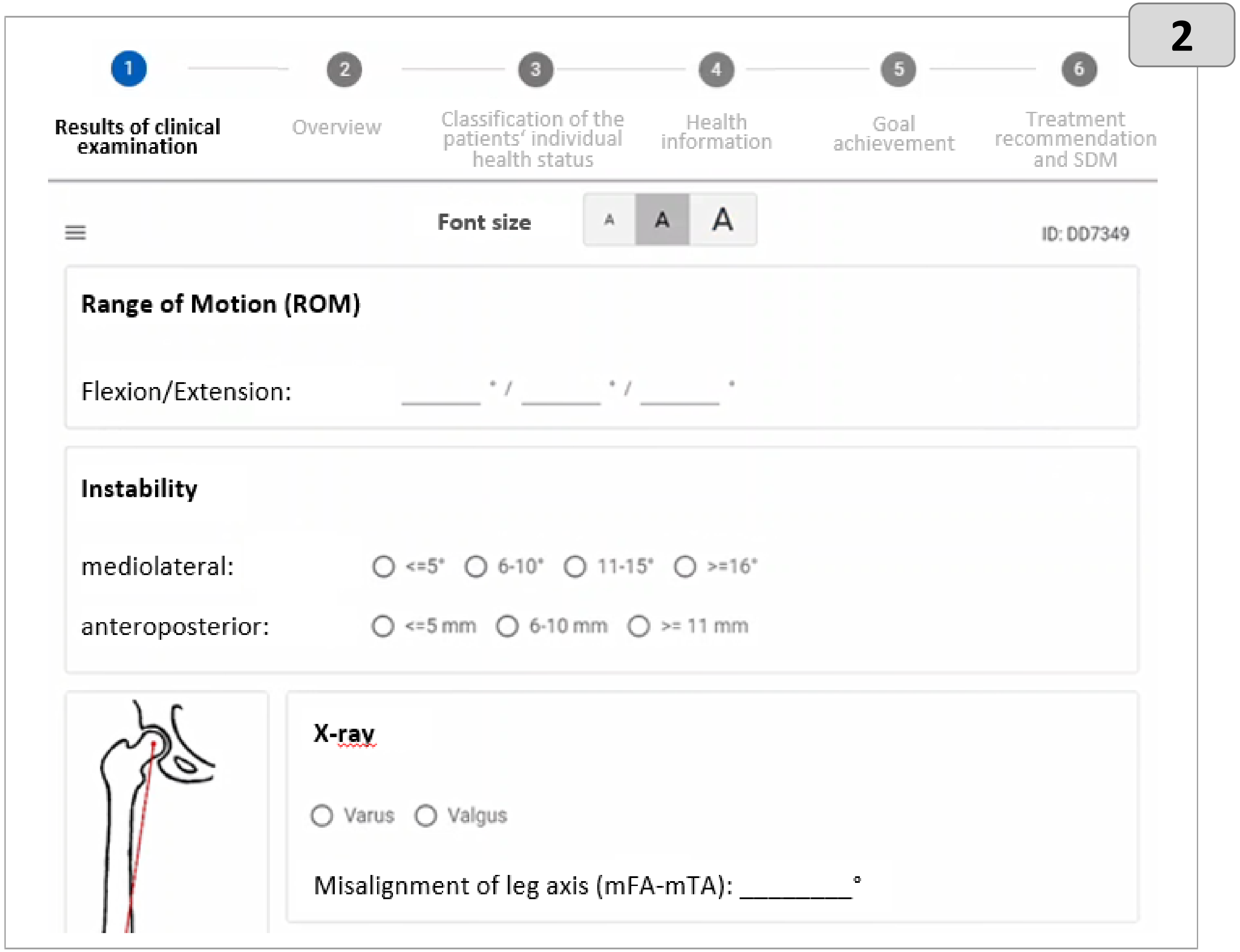

2
1 Results of clinical examination
2 Overview
3 Classification of the patients' individual health status
4 Health information
5 Goal achievement
6 Treatment recommendation and SDM
Font size A A A
ID: DD7349
Range of Motion (ROM)
Flexion/Extension: ______ ° / ______ ° / ______ °
Instability
mediolateral: ◯ <=5° ◯ 6-10° ◯ 11-15° ◯ >=16°
anteroposterior: ◯ <=5 mm ◯ 6-10 mm ◯ >= 11 mm
X-ray
◯ Varus ◯ Valgus
Misalignment of leg axis (mFA-mTA): ________°

PHASE II

ValueBased TKR

Informed SDM through the use of individual operationalized data and health information during TKR consultation

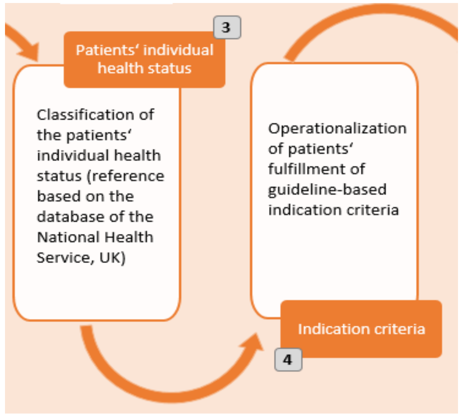

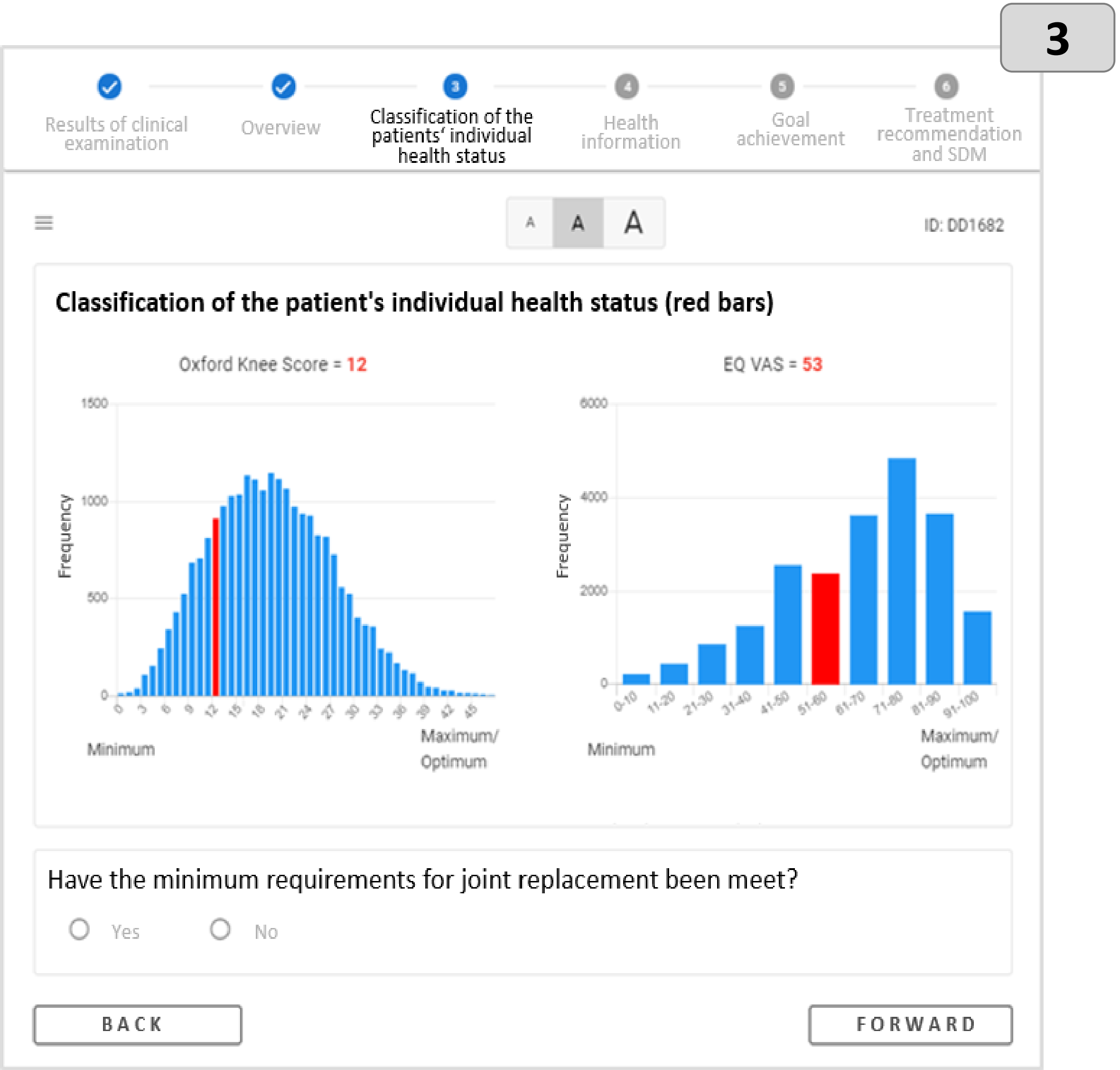

PHASE II

Informed SDM through the use of individual operationalized data and health information during TKR consultation

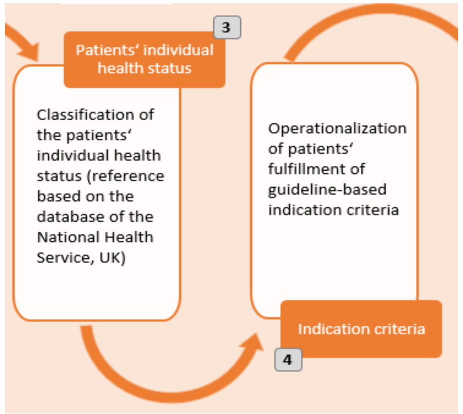

4

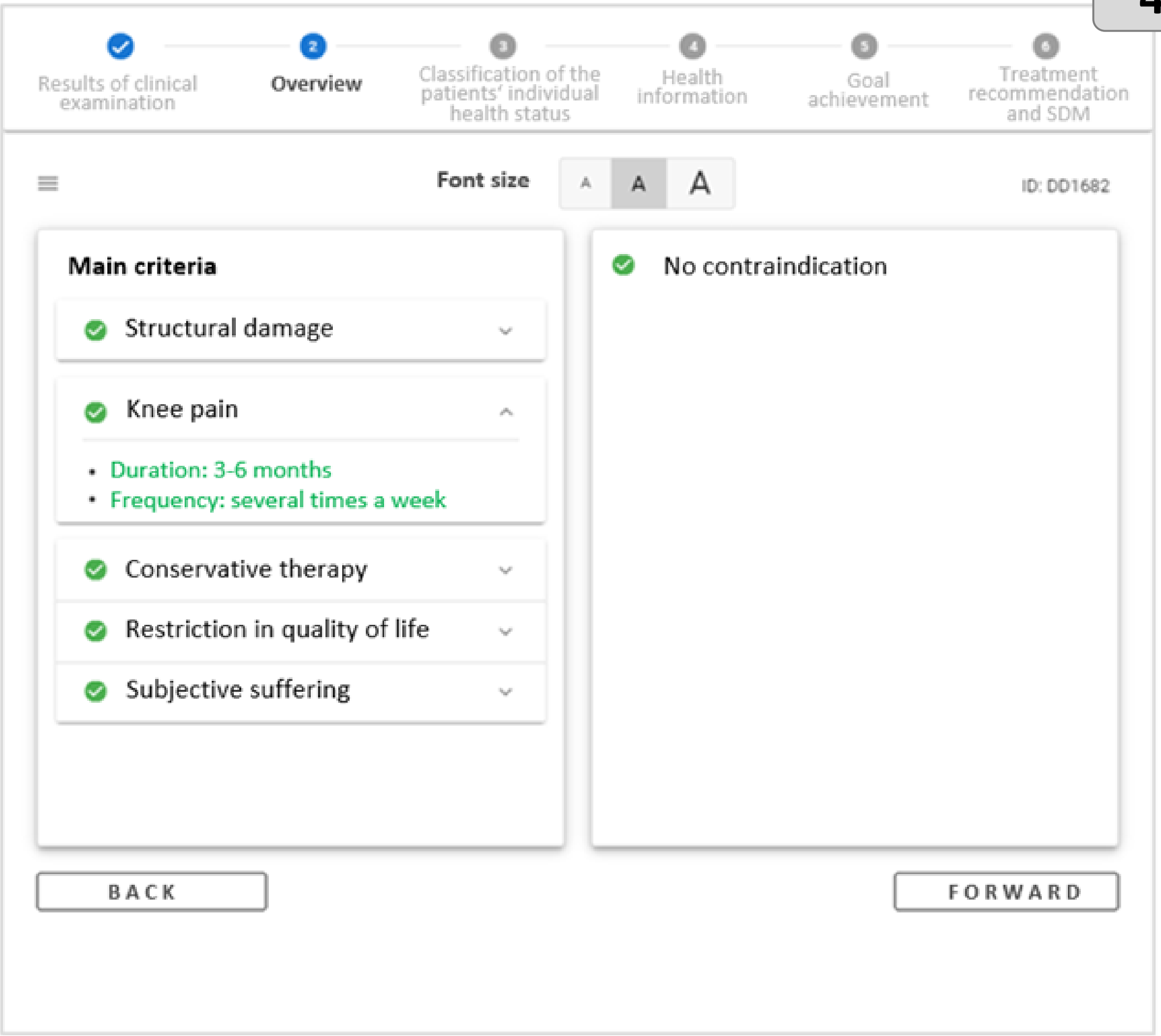

PHASE II

Informed SDM through the use of individual operationalized data and health information during TKR consultation

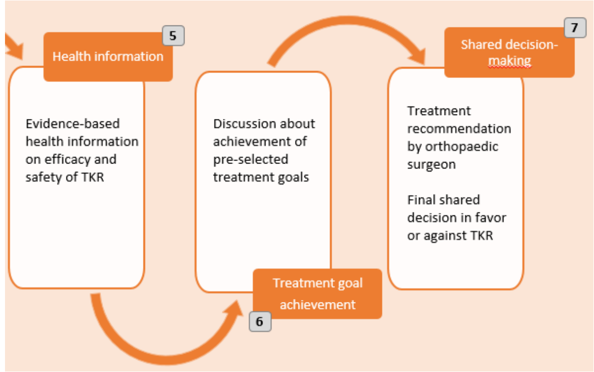

5

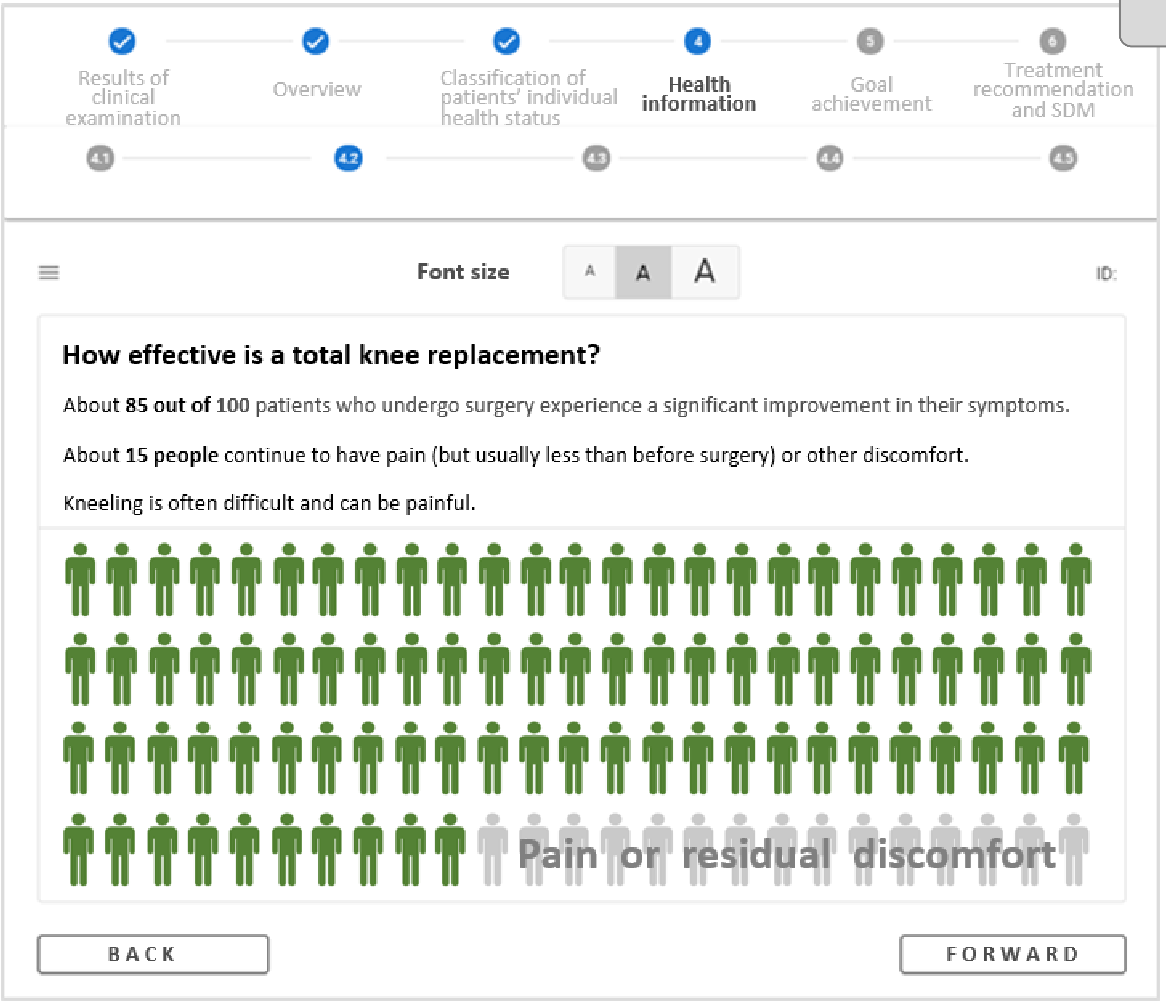

ValueBased TKR

PHASE II

Informed SDM through the use of individual operationalized data and health information during TKR consultation

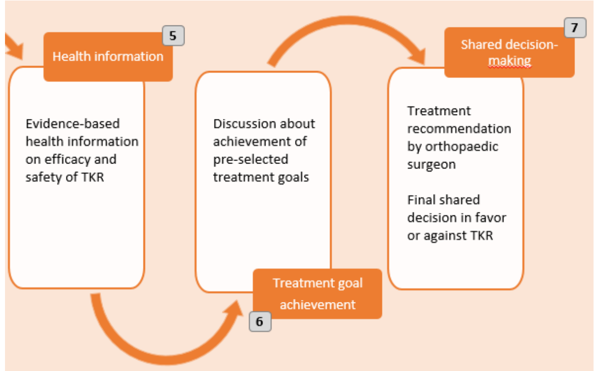

6

Results of clinical examination — Overview — Classification of the patients' individual health status — Health information — **5 Goal achievement** — 6 Treatment recommendation and SDM

Font size A A A — ID: DD1682

For each question, please estimate the degree of likelihood that your patient will achieve the following goals. You will only be shown goals that your patient has previously marked as main goals.

	Very likely	Likely	Rather likely	Rather unlikely	Unlikely
**Symptoms**					
Knee pain relief	◉	○	○	○	○
**Physical function**					
Improving mobility of your knee	○	◉	○	○	○
**Other goals**					
Long lifetime of the artificial joint (duration until a follow-up operation)	○	◉	○	○	○

BACK — FORWARD

PHASE II

ValueBased TKR

Informed SDM through the use of individual operationalized data and health information during TKR consultation

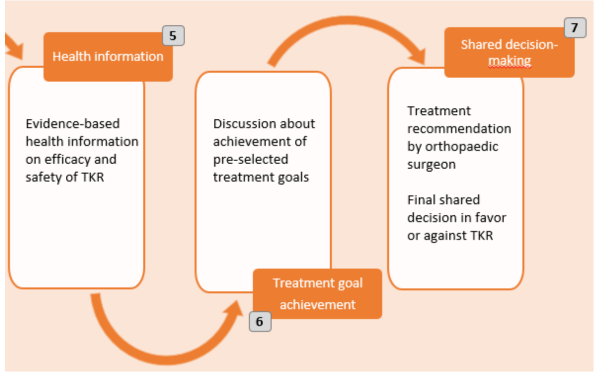

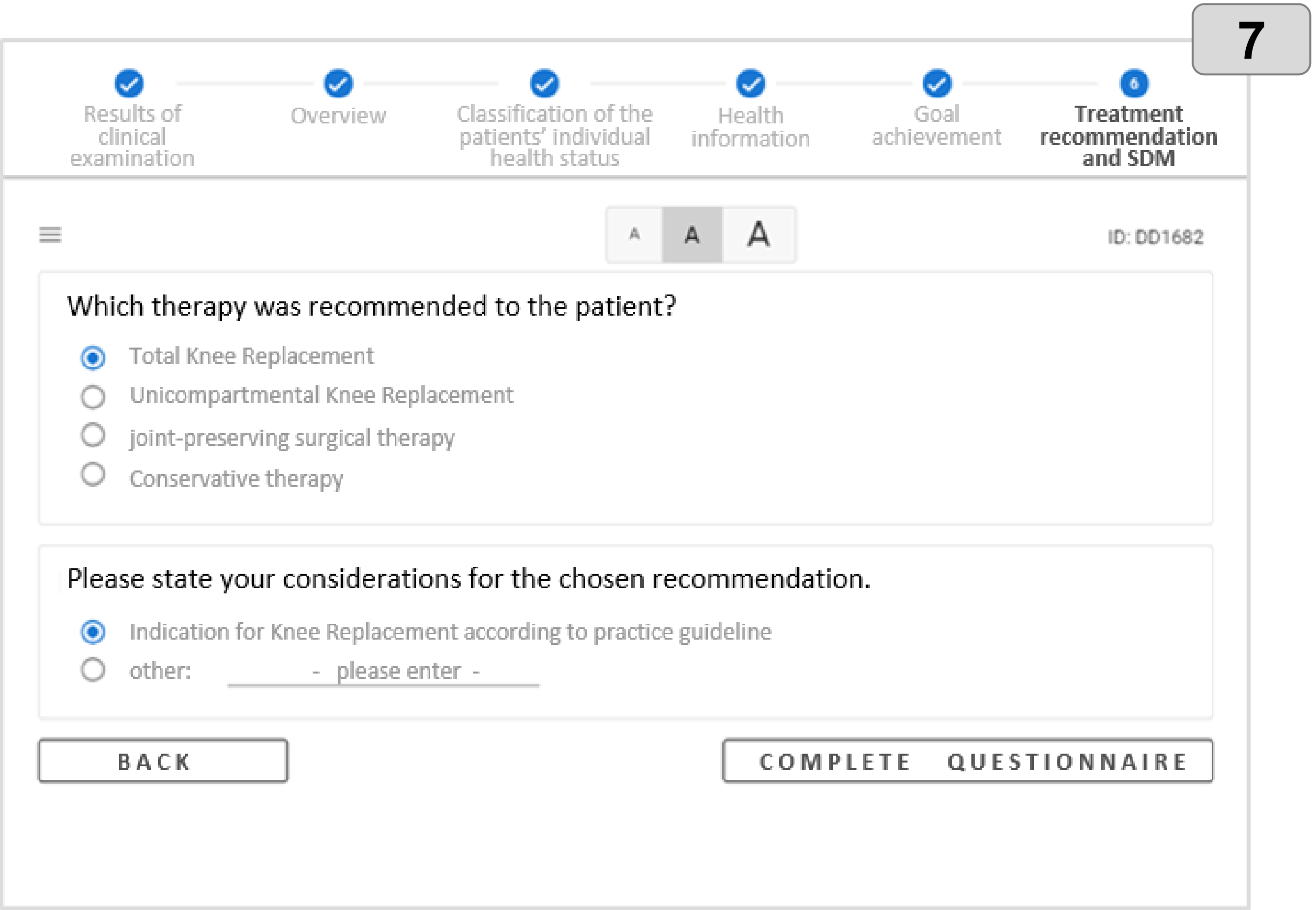

Supplement: Supplementary file 2 — Additional file 2: Screenshots of user interfaces of the EKIT tool. [file 12913_2025_13351_MOESM2_ESM.pdf]
