# Supplementary material for: Facilitators and barriers to the use of a personalised digital decision aid in total knee replacement consultations: insights from patients and orthopaedic surgeons – an interview study
Source: BMC Health Serv Res. 2025 Oct 21;25:1387. doi: 10.1186/s12913-025-13351-y (PMC12541942; doi:10.1186/s12913-025-13351-y)
Supplement: Supplementary file 4 — Additional file 4: Interview guide orthopaedic surgeons. [file 12913_2025_13351_MOESM4_ESM.pdf]

Supplement 6: Coding system patients with knee osteoarthritis and candidates for TKR (MAXQDA)

List of Codes	Frequency of coding	Anchor example for the code
**Code System patients in total**	270	
**Facilitators**	1	
**Benefits of the tool**	0	
Need of information for clinicians/other stakeholders	6	I felt it was necessary so that there are certain things available in the documents. The doctor would have to fill in forms again by hand, that would only cost unnecessary time. Patient2_female_78years, Pos. 257
Positive influence on the decision-making process	0	
Assessment of health status in comparison with others	1	And this is something that I find really interesting, I am in the middle here. This consoles me a little bit, that I'm not quite so bad after all. Patient6_female_69years, Pos. 101
Better assessment of knee complaints	1	So now I know quite well where I'm at. Unlike before, then I thought „I really need to have this surgery". But this showed me that it's not quite this bad yet. Patient6_female_69years, Pos. 153
Promotion of more critical discussion	2	If it [the EKIT-Tool] had been available then [at my previous knee surgery], maybe I might have looked into it more intensively just as a suggestion. Patient6_female_69years, Pos. 135
Dealing with own expectations	2	But from the patients perspective, it's not a bad idea to ask what do you expect from it. From an operation like this, or in general: how do you expect things to continue? Patient3_male_53years, Pos. 51
Positive influence on the decision	0	
Encouragement to wait with surgery	2	This was a confirmation that a prosthesis would definitively have to wait. Patient4_female_54years, Pos. 129
Confirmation of the treatment goal	1	That was actually a confirmation what I had set as a goal myself anyway. Patient3_male_53years, Pos. 81
**Barriers**	0	
**Criticizing the tool**	4	
Statistics/diagrams do not help with the decision	1	This operations are definitive. You can't reverse them. And I say, even if he gives me statistics, that doesn't help me either. Patient4_female_54years, Pos. 141
Main focus on surgery and not on non-operative procedures	5	But there are great aids that can delay this. Also as a support and that was missing. Patient4_female_54years, Pos. 80
**Barrier to filling out the questionnaire (phase I)**	1	
Setting	1	
Full waiting room/lack of quiet space	1	The nice young lady took me into her room, everything was quite crowded, and we filled it out in the doctor's office. Patient7_female_64years, Pos. 35
Time factor	3	Of course, it's easier if you can take two or three minutes to think about it. Patient9_male_60years, Pos. 47
Technology	4	
Handling touch on the tablet	2	But as you get older, your fingers end to become a bit wider, you know. And the circles on the tablet are quite close together. Patient2_female_78years, Pos. 133
On the patient side	0	
Excitement	1	Because yesterday there was also added excitement, and yes. Patient7_female_64years, Pos. 208

Supplement 6: Coding system patients with knee osteoarthritis and candidates for TKR (MAXQDA)

List of Codes	Frequency of coding	Anchor example for the code
Age	3	But I imagined what the patient group is like in my age, s there will probably be problems sometimes. Patient2_female_78years, Pos. 117
Visual impairment	3	With my eyes, with the macula [degeneration]. I look at a point and earlier I didn't see it and now I see it. Patient2_female_78years, Pos. 71
Enter targets	1	I put quality of life and mobility and no pain there. But what ist he main goal now? In principle, of course, it's mobility and then no pain, and if I have both I automatically have a better quality of life. Patient7_female_64years, Pos. 71-74
**Barriers during the conversation with physician (phase I)**	0	
Scope of information depends from person	1	But something like this also really depends on the person. My daughter finds something like this extremely interesting. Patient7_female_64years, Pos. 190
Excitement at the physician's appointment	1	I mean it's always a certain situation, doctor to patient. I mean, when you sit there and say well, it's easy to be a bit more positive or negative. Patient1_male_80years, Pos. 73
Favouring paternalistic decision-making model	1	Otherwise, I honestly believe that I should let myself be guided by the experts, because I have no idea about the matter. Patient8_male_61years, Pos. 164
Lack of specialised knowledge	1	You don't really have the expertise fort hat, I'd say. Patient8_male_61years, Pos. 181
Lack of explanations for illustrations	1	The doctor first told be about the pictures and I don't really know what they mean.
Overwhelmed by the illustrations and explanations	1	I could't have realized that in this moment, I say, because from the other I was, well (…) it's also impressive at first. Patient8_male_61years, Pos. 119
Too "medical" formulations	1	I mean, the names and so on, if it was medical, then not. Patient1_male_80years, Pos. 157
Graphics are superfluous	1	It's actually better explained in three sentences that with all these pictures. Patient8_male_61years, Pos. 29
Subjectively too much explanation/information	2	Sometimes it is just too much information. Patient7_female_64years, Pos. 188-190
**Experience of the application on the tablet (phase I)**	0	
<u>Practical realisation/implementation</u>	0	
No support	4	I: Did you operate the tablet on your own?<br>P: Yes. I operated it alone. P3_m53, Pos. 32-35
Needs support	5	Yes, I was holding it and Ms. [Name] did a really nice job and when I couldn't manage with the fingers. Patient2_female_78years, Pos. 22-25
Complete takeover	12	I: so that means you said the answer and the nurse clicked?<br>P: Yes exactly. Patient7_female_64years, Pos. 68-69
<u>First impression</u>	13	Well, I didn't really have a problem with it. I have to say in this age of modern technology, I don't think it's so bad that it's done like this. Patient3_male_53years, Pos. 31
<u>Scope of time</u>	9	You had time to read everything or imagine it and then I could say to myself, yes, that's how I decide. Patient1_male_80years, Pos. 85

Supplement 6: Coding system patients with knee osteoarthritis and candidates for TKR (MAXQDA)

**Usability of the tool**	0	
Difficult	2	And the circles on the tablet are quite close together, so you always have to have a look that maybe you tap it with the edge of the finger instead of the tip. Patient2_female_78years, Pos. 133
Simple	11	I: How is it for you in terms of usability?<br>P: I don't have a problem with it. Everything went smoothly. No problems. Patient3_male_53years, Pos. 36-39
**Comprehensibility part tablet (phase I)**	0	
<u>Contents</u>	0	
Understandable	13	The questions are understandable, I just need to really think about the question. Patient1_male_80years, Pos. 61
Incomprehensible	4	Right, I overlooked that and probably thought: „I'll have to tap on 10 here again" or something. The question there wasn't clear. Patient2_female_78years, Pos. 36-39
<u>Representations</u>	0	
Understandable	9	It was actually made understandable that I, um, actually shortened the questions there in which box I had to make the cross or the tick. Patient1_male_80years, Pos. 45
Incomprehensible	1	
**Patient-physician conversation (phase II)**	0	
<u>Comprehensibility in the conversation</u>	0	
Contents	0	
Understandable	6	I: Yes, yes okay. Can you recall if you ever had the feeling „Now I don't understand what the doctor is explaining right now."?<br>P: No. Patient4_female_54years, Pos. 93-96
Incomprehensible	1	I mean, the names and so on, if it was medical, then not. Patient1_male_80years, Pos. 157
Representations	0	
Understandable	16	He can show me what it looks like and where the bend is or, um, of course, it's a bit easier to visualise. I thought it was good, I thought it was good. Patient1_male_80years, Pos. 131
Incomprehensible	7	I: Okay and on this other sheet, this categorisation of the current health status. There is this Oxford-Knee-Score/<br>P: Yes, on the left side equals „18" it says. Well, this I didn't understand. This, you see, I wanted to google this yesterday evening. Patient7_female_64years, Pos. 101-102
<u>Satisfacition</u>	0	
Satisfied	16	What did you think of the fact that the computer is now added to the doctor's consultation?<br>P: Well yes, positive, positive. Patient1_male_80years, Pos. 130-131
Dissatified	6	You have to wait a long time to get an appointment in a consultation like this. And I did not think the result was great. Patient4_female_54years, Pos. 161

Supplement 6: Coding system patients with knee osteoarthritis and candidates for TKR (MAXQDA)

**Decision making**	0	
Openness to results	0	
Not open-ended in the dialogue	11	Yes, you look into it a long time in advance, at least I did, and make enquiries. So it wasn't a spontaneous decision on my part. Patient2_female_78years, Pos. 199
Open-ended dialogue	2	In general open. So I try to delay this as much as possible. So I don't really want to do it. Patient3_male_53years, Pos. 26-29
Own part of the decision/SDM	5	
Influence on decision-making	0	
No influence	6	What influence, so no, I don't think so. Whether there's a computer there or a tablet or whatever. It is like that but it has no direct influence on it (..). Patient8_male_61years, Pos. 200-201
Little influence	3	I: So it encouraged you in your decision?<br>P: Yes, exactly. P1_m80, Pos. 153-155
Strong influence	4	So now I know quite well where I'm at. Unlike before, then I thought „I really need to have this surgery". But this showed me that it's not quite this bad yet. Patient6_female_69years, Pos. 153
Certainty with the decision	0	
Uncertain	2	I: How confident are you in the decision that this operation will take place? From your side?<br>P: Wee, you can ask me that again and again every hour and it will change every time, depending on my state of mind. Patient7_female_64years, Pos. 152
Certain	7	I: How confident are you in the decision?<br>P: I am 100% sure. Patient8_male_61years, Pos. 168-171
Satisfaction with the decision	0	
Dissatisfied	1	I: How content are you with it?<br>P: Yes, it's okay for me. I had slightly different expectations of the therapy. Patient4_female_54years, Pos. 61-62
Satisfied	4	I: How content are you with the decision now?<br>P: So at the moment I'm quite satisfied. Or very satisfied. Transcript_Patient3_male_53years, Pos. 66-67
**Perceived benefit**	0	
No benefit	2	It doesn't usually do much for the patient. Whether I do this or not, it doesn't do me any good. If I say 'I can no longer climb stairs' and click on it, then I know that either way, whether I have clicked on it or not. Patient9_male_60years, Pos. 169
Little benefit	3	I: And did you enjoy using this in the decision-making process?<br>P: I felt it was necessary so that there are certain things available in the documents. The doctor would have to fill in forms again by hand, that would only cost unnecessary time. Patient2_female_78years, Pos. 256-259
Great benefit	7	I would have been happy if this had been available with my first knee already. Patient6_female_69years, Pos. 166-168
Recommendation	3	
To another person	8	Yes, definitely. I have already advertised it, I have to say. Because there are quite some people, some have problems here, others there. Patient6_female_69years, Pos. 169-172

Supplement 6: Coding system patients with knee osteoarthritis and candidates for TKR (MAXQDA)

For hip arthroplasty	4	I: Alright. Assuming you needed a new hip, could you imagine using it in a similar way? So<br>P: I would do it the exact same way.<br>I: The exact same way, so again the questionnaire on the tablet and/<br>P: Yes.
