# Supplementary material for: Facilitators and barriers to the use of a personalised digital decision aid in total knee replacement consultations: insights from patients and orthopaedic surgeons – an interview study
Source: BMC Health Serv Res. 2025 Oct 21;25:1387. doi: 10.1186/s12913-025-13351-y (PMC12541942; doi:10.1186/s12913-025-13351-y)
Supplement: Supplementary file 5 — Additional file 5: Data coding system patients with knee osteoarthritis and candidates for TKR. [file 12913_2025_13351_MOESM5_ESM.pdf]

## Supplement 5: Coding system orthopaedic surgeons (MAXQDA)

| List of codes | Frequency of coding | Anchor example for the code |
| --- | --- | --- |
| **Code system physician in total** | 282 | |
| **Faciliators** | 47 | |
| **Benefits of the tool** | 0 | |
| **Improving communication** | 0 | |
| Improvement of the post-operative situation | 1 | |
| Reduction of dissatisfied patients | 1 | If discussions are necessary because the patient is dissatisfied, then that takes time. And it is much more difficult to talk to a dissatisfied patient than it is to talk to them before (laughs) when they simply have too high expectations. (OS1_female aged 36, Pos. 130-135) |
| Facilitate communication with low health literacy | 1 | I would say that the tool would be quite good for people who are a bit simple-minded, because you often notice that they have difficulty with open questions, in the sense that they don't understand the direction the question is going in. The points that are asked about are helpful in getting a clear answer. (OS8_male_age not reported, Pos. 72) |
| Realistic categorisation of expectations | 4 | And since the expectations are easy to sort out realistically, that makes it easier for me. (OS1_female aged 36, Pos. 122) |
| Patients have a chance to say/ask more | 2 | The only thing that has changed is that patients now have the opportunity to say more. (OS1_female aged 36, Pos. 118) |
| Better communication about patient goals/expectations | 8 | I find that very helpful. Most patients come in and say, “I want to play tennis again,” or “Golf is important to me again,” or “I want to do this or that,” and then you can explain whether that's possible or not. (OS7_male aged 54, Pos. 146-148) |
| **Positive influence on the decision-making process** | 0 | |
| Decision/indication is specified | 1 | In the decision-making process, I think about it a little more precisely. (OS7_male aged 54, Pos. 54) |
| Standardisation/structuring of the consultation/ conversation | 7 | I think that, overall, this could lead to patients being informed about the risks and benefits in a more standardized way. I think that's a good thing. (OS6_male aged 36, Pos. 34) |
| Clarification of benefits/risks for patients | 7 | This visualization makes it more tangible for them than if I were to draw the complications on the surgical information sheet, so to speak. (OS5_female aged 45, Pos. 49) |
| Scientific approach | 1 | You just talk about the whole thing in a much more scientific way [with the EKIT tool]. (OS2_male aged 39, Pos. 49) |
| Patient feels perceived „differently“ | 1 | I think patients also feel that they are perceived very differently when they disclose certain things. (OS1_female aged 36, Pos. 63) |
| More realistic assessment of the initial situation before surgery | 1 | I think they were able to comprehend their own image a little better. (OS4_female aged 26, Pos. 35) |
| Self-assessment of state of health/goals | 3 | Because, at one point, patients assess their own state of health, and this sometimes differs from what you might gather in a normal conversation or think patients are thinking. (OS1_female aged 36, Pos. 3) |
| Provides food for thought for the patient | 2 | The fact is that you first have to initiate a thought process about the risks and side effects and the chances of success. (OS7_male aged 54, Pos. 54) |
| Revealing and clarifying decision-making conflicts | 1 | Yes, I think so, especially since conflicts that were previously unknown are now being uncovered. (OS1_female aged 36, Pos. 67-69) |
| **Positive influence on decision** | 0 | |
| More active involvement of patients | 1 | Yes, I think so, because you make decisions together with the patient, so you involve them in the decision-making process in every way. I don't think that happens as much without it.. (OS1_female aged 36, Pos. 48) |

## Supplement 5: Coding system orthopaedic surgeons (MAXQDA)

| List of codes | Frequency of coding | Anchor example for the code |
|---|---|---|
| Confirmation of the indication | 1 | It's also a kind of safeguard. It may well be that I have the X-ray image, which shows me where I say the indication is the patient's restricted mobility, the level of suffering, and the tool also provides confirmation of the indication based on the stored data. (OS5_female aged 45, Pos. 76) |
| Additional confirmation of the decision | 2 | As I said, many people were convinced by the fact that they had been asked and that the tool had also indicated that the indication was there. You could see that some people thought, “Oh, he says so too, that’s good.” (OS5_female aged 45, Pos. 106) |
| Greater certainty with the decision | 1 | I already had that feeling AFTER the survey, the thing was different. At the beginning, they were more uncertain than at the end. (OS4_female aged 26, Pos. 89) |
| **Barriers** | 114 | |
| **Criticising the tool** | 0 | |
| Absence of risk factors/comorbidities | 8 | I don't know if you can say that it's better to enter risk factors like that. Because with infections and things like that, it's clear that I won't operate. (OS3_male aged 43, Pos. 113-115) |
| Lack of openness to results on the part of the patient | 14 | But most of them had already decided to have the operation. And that's why not much has changed. Perhaps she confirmed that, or yes. (OS3_male aged 43, Pos. 63-64) |
| Indication of the examination findings flexion and extension | 1 | Once I entered it, that was the initial problem. I didn't enter ‘flexion, extension’ as it was written there, but did it exactly the other way around and was surprised that he complained about it. (OS5_female aged 45, Pos. 149) |
| Already extensive/direct information consultation | 1 | |
| Advance performance up to graph | 1 | I've seen that before. But until you see this graph, so to speak, you have to make an advance payment, if you will. And that's what she did; the advance payment often bothered her quite a bit. (OS2_male aged 39, Pos. 45) |
| Awareness of the surgical risk is negative | 1 | |
| Illustration of the "healthy" knee replacement patient | 1 | The tool essentially depicts a healthy patient with a total knee replacement and can show them that everything can go well. However, there are also 20 percent of patients who, even though everything is perfect, are not satisfied. (OS6_male aged 36, Pos. 110) |
| Misunderstandings with patient details | 2 | We noticed that we hadn't thought everything through when marking the boxes in advance, and when I asked about specific points, there was indeed something there. (OS1_female aged 36, Pos. 77) |
| Relatively detailed knowledge | 1 | Relatively detailed knowledge. (OS7_male aged 54, Pos. 142) |
| Meaningfulness of the absolute contraindications | 1 | It's clear to me now that I won't install a prosthesis if there's an infection or if he only has three months to live. I would just ignore it. (OS3_male aged 43, Pos. 79-80) |
| Repetitions in the tool | 3 | And there is another graph similar to this one, where the age of the patients is also indicated. Every time I see it, I notice that I say that it is a similar image, a similar representation, and I think that the repetition may not be necessary. (OS1_female aged 36, Pos. 15) |
| **Barriers to filling out the questionnaire (phase I)** | 0 | |
| Technical factors | 5 | Exactly, and then we just logged out, restarted, and then it was fine. That's why I made sure to check that I had entered everything right when I was typing it in, so that I didn't have to correct anything. (OS5_female aged 45, Pos. 117-118) |
| Time factor | 4 | |
| Organisational processes of the clinic/practice | 3 | They have to ask the patient if he wants to participate. Then she has to explain the tablet to him. It's just too much. We don't have that much time in the practice. (OS7_male aged 54, Pos. 17) |
| Patient-related factors | 0 | |

## Supplement 5: Coding system orthopaedic surgeons (MAXQDA)

| List of codes | Frequency of coding | Anchor example for the code |
|---|---|---|
| *Technical experience* | 4 | Not everyone was able to use the tablets properly. (OS4_female aged 26, Pos. 11) |
| *Cognitive impairments* | 3 | That wouldn't have been suitable for the patient. She has a carer who signs for her, and I don't think she can read properly herself, or only a few words. It wouldn't have worked with her. (OS1_female aged 36, Pos. 83) |
| *Visual impairments* | 1 | or they couldn't read it because the font was too small. (OS4_female aged 26, Pos. 13) |
| *Age* | 7 | Are there patients who are unsuitable for using the tool? Or for whom its use is at least difficult? Ä: Yes, I would be critical of anyone over the age of eighty. (OS5_female aged 45, Pos. 63-64) |
| *Language barriers* | 3 | Then there is the language barrier, patients who do not speak the language very well. (OS3_male aged 43, Pos. 71) |
| *Excitement* | 1 | They're super excited, and then something like this happens. (OS2_male aged 39, Pos. 98) |
| *Need for personal support* | 5 | Yes, but I don't know what influence his relatives will have, because the goal is for him to do it on his own. (OS1_female aged 36, Pos. 140-142) |
| **Barriers during the conversation (phase II)** | 2 | |
| Physician-related factors | 0 | |
| Time factor | 5 | |
| *Extension due to explanations* | 3 | And the conversation is prolonged solely because I then have to explain this. (OS2_male aged 39, Pos. 19) |
| *Too extensive explanations of graphs/scores, text* | 5 | Exactly, that's what I say too, for example, but if I now have a graph, I have to explain everything that can be seen there. I have to explain a curve, explain the colours, and that makes it longer. (OS2_male aged 39, Pos. 21) |
| *Discussing too many goals* | 5 | So, the only thing is, of course, there are patients who enter 30 goals, which is laborious (laughs), because each individual goal has to be discussed. (OS1_female 36, Pos. 177) |
| Lack of knowledge | 0 | |
| *Lack of technical skills* | 1 | My colleague is 55 years old. He had considerable problems clicking through the tool and doing this. (OS6_male aged 36, Pos. 129) |
| *About the scores* | 1 | They don't know what the Oxford Knee Score is. Or even the EQ 5D, they simply don't know it, they don't work with it. Then, of course, it's harder to get into it and explain it well to patients. (OS1_female aged 36, Pos. 169) |
| patient-related factors | 0 | |
| Patient characteristics | 10 | |
| *Age* | 4 | They are also overwhelmed by it, to some extent. I mean the elderly, those with mild dementia, who are a little confused due to their age. It's too much for them. (OS6_male aged 36, Pos. 68) |
| *Hearing/language barrier* | 2 | Well, I remember having patients over the past two or three years who were deaf and non-speaking. From our side, it was extremely difficult to communicate and convey information in those situations. |
| *Excitement* | 3 | People go to the doctor and are extremely anxious, and then it's a question of whether they need surgery or not. (OS2_male aged 39, Pos. 45) |
| Different levels of knowledge | 2 | |
| Needs of the patient | 1 | |
| *Excessive demands on the patient* | 2 | If you then start confronting them with all sorts of things, and perhaps even with science, I think they will really be overwhelmed. I don't think you're doing them any favours that way. (OS7_male aged 54, Pos. 73) |

## Supplement 5: Coding system orthopaedic surgeons (MAXQDA)

| List of codes | Frequency of coding | Anchor example for the code |
|---|---|---|
| *No request for risk disclosure* | 4 | You often hear people say, 'Oh God, I don't want to know all the details! Where do I sign?' That's very, very common. (OS8_male age not reported, Pos. 68) |
| *No preference for SDM* | 1 | There are patients who want to be guided a little. Imagine that they don't necessarily want to decide for themselves. And they say, let the doctor do it. He's the specialist, after all. (OS7_male aged 54, Pos. 73) |
| *Other influencing factors predominate* | 2 | Because they wanted to focus on other matters. They are so excited that they do not want to fill out any forms or click through anything at this point. (OS2_male aged 39, Pos. 45) |
| **Usability** | 0 | |
| Difficult | 1 | I had the experience once with Ex and Flex, which is different in the EKIT tool than how I write it down, and when I got an error message like that, I sometimes had the problem that I then had to go back to the correction, which somehow hadn't been accepted. (OS5_female aged 45, Pos. 116) |
| Easy/simple | 17 | I: Did you personally find it easy or difficult to use the EKIT tool?<br>A: No, it was easy for me. But I am a member of the younger generation. So it worked, it was really easy. (OS4_female aged 26, Pos. 20-21) |
| **Comprehensibility of the tool** | 0 | |
| Contents | 0 | |
| Understandable | 7 | They sometimes ask questions when they don't understand something, but everything can be clarified easily. There aren't really any obstacles. (OS1_female aged 36, Pos. 18-21) |
| Incomprehensible | 0 | |
| Representations | 1 | |
| Understandable | 15 | You can see that quite clearly there. It's also illustrated really well with the graphs, if you look at them. For example, if you look at patient satisfaction and the likelihood of revision and all that. It's illustrated very well. (OS2_male aged 39, Pos. 25) |
| Incomprehensible | 4 | The only thing for most people, well, for me, the EKIT tool wasn't really a problem, but the font was very small. (OS4_female aged 26, Pos. 160) |
| **Patient-physician conversation (phase II)** | 0 | |
| Evaluation of communication | 0 | |
| Worsened | 2 | So, for those who are technically savvy, I find it difficult because it raises additional questions that then disappear into the small print. (OS5_female aged 45, Pos. 100) |
| Neutral/no change | 3 | I: (...) Does that make communication with the patient more difficult or more easy?<br>Ä: It depends. (OS5_female aged 45, Pos. 99-100) |
| Improved | 9 | But of course, this is much better because it is based on the information provided by the patient and not just what is on my checklist. (OS1_female aged 36, Pos. 63) |
| Time expenditure | 0 | |
| Reduced/postponed | 3 | A: And it's much more difficult to talk to a dissatisfied patient than it was before (laughs) when they simply had unrealistic expectations.<br>I: Yes, OK. So maybe it's not extra time, but rather time that has been shifted in the process.<br>A: Exactly. (OS1_female aged 36, Pos. 130-134) |
| Same | 2 | So sometimes they ask more questions and sometimes less. But that has nothing to do with the software or the tool. (OS2_male aged 39, Pos. 9-11) |

## Supplement 5: Coding system orthopaedic surgeons (MAXQDA)

| List of codes | Frequency of coding | Anchor example for the code |
|---|---|---|
| Increased | 13 | So that means for the standard patient, I would say, it took longer for me. (OS3_male aged 43, Pos. 24) |
| **Decision making** | 0 | |
| Influence | 0 | |
| Influence present | 4 | So I think, for me, that means when I do this in practice, during the decision-making process, I think about it a little more carefully. (OS7_male aged 54, Pos. 54) |
| No change | 5 | To be honest, this has not changed my decision-making process. (OS2_male aged 39, Pos. 43) |
| Facilitated | 5 | Specifically, in response to the question, yes, it may have made the decision easier. (OS4_female aged 26, Pos. 25) |
| More difficult | 1 | But if the patient comes to me for surgery and someone else has already been involved, it might confuse them. Then it might be more difficult to decide for or against it. (OS7_male aged 54, Pos. 56) |
| **Perceived benefit** | 0 | |
| Little benefit | 3 | I believe it has the potential to be an added value. Unfortunately, I can't say that yet because I've only used parts of the tool. (OS8_male age not reported, Pos. 129-130) |
| Great benefit | 14 | And if you have a tool like this to help you, it's certainly very useful. Because even I don't always think of everything. (OS7_male aged 54, Pos. 148) |
| Recommendation | 0 | |
| To colleagues | 6 | I: And would you recommend the use of this tool to a colleague?<br>A: Well, if the study has resulted in improvements and optimisations, then I can certainly imagine doing so. (OS3_male aged 43, Pos. 118-119) |
| For hip arthroplasty | 6 | Could you imagine the EKIT tool now also being adapted for use in other surgeries, such as hip replacements or similar procedures?<br>A: Yes, absolutely. Yes, definitely. (OS2_male aged 39, Pos. 101-102) |
