# Supplementary material for: Facilitators and barriers to the use of a personalised digital decision aid in total knee replacement consultations: insights from patients and orthopaedic surgeons – an interview study
Source: BMC Health Serv Res. 2025 Oct 21;25:1387. doi: 10.1186/s12913-025-13351-y (PMC12541942; doi:10.1186/s12913-025-13351-y)
Supplement: Supplementary file 6 — Additional file 6: Data coding system orthopaedic surgeons. [file 12913_2025_13351_MOESM6_ESM.docx]

| **First impression and “ice breaking” questions** |
| --- |
| 1. How long have you been suffering from knee pain? 2. How long have you been receiving medical treatment due to your knee pain? 3. What prompted you to make an appointment at the orthopedic clinic/center to discuss about the pros and cons of a total knee replacement (TKR)? *(Have you been referred by a physician, was it your own choice or was this a decision after consulting your general practitioner?)*   You have made an appointment at the orthopedic clinic/center for TKR consultation aiming to discuss about the possibility of a TKR:   1. How much time has passed since this appointment? Was this your first consultation regarding (surgical) treatment options due to your knee pain/knee osteoarthritis (knee OA)? |
| **PHASE I**  **Using a tablet *prior* TKR consultation** |
| You received a tablet before starting the TKR consultation and were asked to provide some personal and disease-specific information beforehand *[reference on screenshots]*:   1. What were your thoughts about that? (about receiving a technical device/aid) What was your first impression? 2. Who entered the data via tablet? *(if the patient was (partly) assisted, ask for the reasons)*   *If the tablet was used (partly) by the patient him/herself:*   1. Was it difficult for you to use the tablet or not? *(if not – please explain)* 2. Did you feel confident using the tablet or not? *(if not – please explain)* 3. Did you consider the tablet as useful in order to collect your personal and disease-specific data? 4. Would you have preferred to use a paper questionnaire? 5. How satisfied are you with using this tablet? 6. Is there something we can do better with our tablets?   *Continued on the next page* |
| **Convenience of using a tablet** |
| Comprehensibility of displays / figures / terms / symbols – *(reference on screenshots)* |
| Please take your time looking at the screenshots of the EKIT tool to remember correctly!   1. Were the displays and figures understandable for you or not?   If not, what exactly was difficult for you to understand? Can you explain why it was difficult for you?   1. Were the terms and symbols you saw on the tablet understandable for you or not?   If not, please tell (or show) me, which terms and symbols were difficult for you to understand? Can you explain why it was difficult for you? |
| **Presentation of the content on the tablet [reference on screenshots]**  Please take your time looking at the pictures! Feel free to mention any little detail that bothers to you! |
| 1. What do you think about the presentation of the content (size of displays, font size, and color contrast)? |
| **Rating the content / functional range of the tablet** |
| 1. How did you appraise the functional range? 2. Would you consider a tablet as too complicated for the advantage it is featuring or do you think it is reasonable? *Please describe more detailed.* |
| **User-system-interaction / handling the tablet** |
| 1. How would you judge handling the tablet in general? 2. Was it rather easy or difficult to navigate to the topics important to you?   DIFFICULT – What exactly made it difficult for you? Please explain.   1. How did you rate the required effort to put in your data into the tablet? 2. Were the tools information to you rather understandable or not? [*reference on screenshots]*   NOT UNDERSTANDABLE - Can you remember which information was/were particularly difficult for you to understand and why?   1. How well did you cope with the tools’ reaction to accidentally incorrect data entries?   *Continued on the next page* |
| **PHASE II**  **Computer-assisted TKR consultation – the decision-making** |
| The next few questions relate directly to the consultation with your orthopedist about the decision-making for or against a TKR:   1. Did you finally decide for or against TKR? If not, what were the reasons? 2. Was it a distinct decision for you? What were the arguments/concerns in favor or against a TKR? 3. Did you understand the reasons for this decison? 4. What part did you had in the decision-making? Are you happy about that? Did you miss anything? What did you like, what was not good? 5. How sure are you with your choice? 6. How happy are you with your choice? 7. Do you think every question/topic have been answered/considered? 8. Have you been able to respect each of your considerations with your decision towards this surgical procedure? Was it rather easy or difficult for you?   **You said there will be a/no total knee replacement due to your knee OA. We’d like to know how you came to this decision: you and your orthopedist used a computer during the consultation and decision-making.**   1. How did you feel about that? (about having a technical aid for the decision-making?) What was your first impression? 2. Did you felt rather confident using the visualisations and information by the computer or uncertain?   UNCERTAIN – What made you uncertain? What do you think needs to be changed in order to decrease insecurity?   1. How satisfied are you with using the computer during the consultation? 2. What impact had the use of the computer on your decision-making? 3. Did the use of a computer helped making the decision for (or against) the knee replacement or was it rather hindering (e.g. because of too much information). [please consider screenshots] |
| **Evaluation the communication between patient and orthopaedic surgeon** |
| 1. How did you felt the communication with your orthopedist while using the computer? Was the usage helping or hindering the communication? 2. Did your orthopedist explained the visualisations and information on the screen to you? Each step? Were you able to understand his or her recommendation for or against a TKR? 3. What do think is needed for a successful doctor-patient communication? What are your wishes? 4. How beneficial do you consider the computer (the tool) during a TKR consultation? 5. Overall, how satisfied were you with your TKR consultation and the decision-making? 6. How satisfied were you with risk-benefit evaluation during consultation? |
| **Benefit / impact of EKIT tool during TKR consultation** |
| 1. Do you think the usage of a tablet and a computer (EKIT tool) had an impact on your decision about a TKR?   YES – Can you describe the impact more detailed?  NO – Why do you think so? |
| **Overall opinion about using the EKIT Tool** |
| 1. Altogether, would you say you had good experiences using a tablet and computer (the EKIT tool) for your decision-making? 2. Would you consider using a tablet and a computer (EKIT tool) reasonable? |
| **Repeated use / recommendation of the EKIT tool** |
| 1. Can you imagine using the tablet or a computer (the EKIT tool) for another surgical decision (e.g. total hip replacement)? 2. Would you recommend the EKIT tool to a friend of yours, who is also facing a (surgical) treatment decision? 3. Would you prefer to have all the information on paper? 4. Would you have needed more time to read and consider all the information? What do you think about completing the survey at home beforehand (on paper or online)?   *Continued on the next page.* |
| **Final question** |
| Finally, I would like to ask a more general question.   1. Which technical devices do you already use every day at home or in your leisure time? *(e.g. digital health applications, blood pressure- or blood glucose meter, smartphone, etc.)* |
